# Spatial Transcriptomic Characteristics of the Aging Human Ovary

**DOI:** 10.1111/acel.70288

**Published:** 2025-11-17

**Authors:** Meiling Zhang, Fanghao Guo, Qing Zhang, Qianhui Hu, Di Sun, Yongjian Ma, Yanquan Li, Mengxi Guo, Haixia Ding, Ying Guo, Baicai Yang, Songmao Li, Ningxia Sun, Yuxuan Zheng, Wen Li

**Affiliations:** ^1^ Center for Reproductive Medicine & Fertility Preservation Program, International Peace Maternity and Child Health Hospital School of Medicine Shanghai Jiao Tong University Shanghai China; ^2^ Shanghai Key Laboratory of Embryo Original Disease Shanghai China; ^3^ NHC Key Laboratory of Medical Embryogenesis and Developmental Molecular Biology & Shanghai Key Laboratory of Embryo and Reproduction Engineering Shanghai China; ^4^ Department of Reproductive Medicine Second Affiliated Hospital of Naval Medical University Shanghai China; ^5^ Human Phenome Institute, Pudong Hospital, Fudan University Shanghai China

## Abstract

Ovarian aging is a complex process that compromises fertility and elevates the risk of reproductive disorders. To elucidate its spatiotemporal dynamics, we integrated single‐nucleus RNA sequencing and spatial transcriptomics to construct a comprehensive aging atlas of 12 human ovarian tissues spanning ages 12–54 (prepubertal, age 12, *n* = 1; young, ages 23–29, *n* = 4; middle‐aged, ages 32–34, *n* = 2; and older‐aged, ages 42–54, *n* = 5). Our analysis revealed aging‐related transcriptomic shifts, including impaired mitochondrial oxidative phosphorylation and reproductive structure development in aged human ovaries. We identified a novel endothelial cell (EDC) subtype, *CLDN5* ^+^ blood EDCs, which exhibited unique functional specialization as semiprofessional antigen‐presenting cells. In contrast to other cell types that lost cell identity during aging, *CLDN5* ^+^ blood EDCs displayed transcriptomic sensitivity to aging, characterized by enhanced antigen‐presenting capabilities, and heightened inflammatory activity. Spatial mapping further uncovered immunoglobulin‐expressing (*IGHG1* ^+^/*IGKC* ^+^) cell accumulation in the ovarian periphery, correlating with advancing age. Critically, aging disrupted global cellular connectivity while amplifying the *DLK1:NOTCH3* axis between theca cells and *CLDN5* ^+^ blood EDCs, which may contribute to the dysregulation of ovarian functions. We also detected the upregulation of *DLK1* in granulosa cells from patients with primary ovarian insufficiency. This study significantly enhances our comprehension of the underlying mechanisms of human ovarian aging and concurrently pinpoints potential therapeutic avenues for addressing related disorders.

## Graphical abstract

Troughing constructing a comprehensive spatiotemporal aging atlas on young and aged human ovaries, we revealed distinct cell types, cellular interactions, and aging‐associated gene expression changes due to aging. Specially, we detected an increase in *DLK1:NOTCH3* expression which may contribute to the dysregulation of ovarian functions.

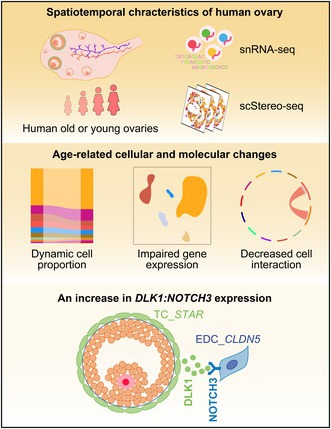

## 1 Introduction

The ovaries play a vital role in maintaining female reproductive health by supporting fertility and hormone secretion throughout the woman's reproductive lifespan (Baerwald et al. 2012; Johnson and Tough 2017). Ovarian function reaches its peak between 20 and 30 years of age, begins to decline thereafter, and typically ceases around 50 (Secomandi et al. 2022; Dong et al. 2023). As the ovary ages, the local microenvironment undergoes changes, leading to a decline in oocyte quality and an acceleration of follicular depletion, ultimately culminating in menopause (Shen et al. 2023; Reiter et al. 2023). The onset of menopause is accompanied by a range of detrimental outcomes, including osteoporosis, cardiovascular disease, obesity, tumors, Alzheimer's disease, and diabetes (Nair et al. 2021; Dalal and Agarwal 2015). Therefore, gaining a deeper understanding of the mechanisms underlying ovarian aging is essential for extending female fertility and mitigating the onset of age‐related chronic diseases (Umehara et al. 2022; Zhou et al. 2024).

Developing effective therapeutic strategies to delay ovarian aging necessitates a comprehensive understanding of the cellular components, molecular properties, and their spatiotemporal changes (Jones et al. 2024; Wu et al. 2024). Recent single‐cell RNA sequencing (scRNA‐seq) and single‐nucleus RNA sequencing (snRNA‐seq) studies have successfully identified various cell types in primate and human ovaries, including the granulosa cell (GC), oocyte (OO), stromal cell (SC), and immune cell (IC) (Wang et al. 2020; Fan et al. 2019; Jin et al. 2024). Recent researches have explored the potential role of perivascular cells in supporting folliculogenesis in ovary health and disease (Xu et al. 2022; Li et al. 2022). Studies have reported an increase in markers of cellular senescence and fibrogenesis within the ovarian stroma with aging (Briley et al. 2016; Ansere et al. 2021), although the specific cell types that become senescent and/or profibrotic remain unknown. Additionally, the ovarian stroma accumulates multinucleated giant cells with age, which may be related to the mechanisms promoting these phenotypes (Foley et al. 2021). A single‐cell atlas in primates found differentially expressed genes (DEGs) in the aged ovarian endothelial cell (EDC), including *CD74*, *FOS*, *FOSB*, *MYC*, and *EGR1*, which were linked to immunoregulation and stress responses (Wang et al. 2020).

The human ovary exhibits significant spatial heterogeneity between the cortex and medulla, with oogenesis initiating in the cortex and substantial restructuring occurring in the medullary region (Zheng et al. 2014; Wagner et al. 2020). While some in situ hybridization (ISH)‐based methods have provided spatial information, they are limited to detecting a few known target genes simultaneously (Chen et al. 2015; Eng et al. 2019). Spatial transcriptomics (ST) captures mRNA from tissue sections using barcoded oligo‐dT primers, enabling spatial visualization of gene expression (Salmen et al. 2018). The single‐cell spatial enhanced resolution omics sequencing (scStereo‐seq) is a novel and powerful spatial RNA sequencing method with nanoscale sensitivity and a large field of view (Chen et al. 2022). Recently, several related studies on ovarian aging using ST have been conducted, including one utilizing the 10× Visium platform (chip size: 6.5 × 6.5 mm) (Wu et al. 2024) and the other utilizing NanoString's GeoMx platform for ovaries from two premenopausal donors of reproductive age (Jones et al. 2024). While these ST technologies are rooted in sequencing or imaging strategies, scStereo‐seq (chip size: 10 × 10 mm) uniquely covers the entire ovarian tissue with a higher resolution in deciphering spatial information, an advantage not feasible with 10× Visium.

In this study, we leveraged snRNA‐seq and scStereo‐seq to investigate the impact of aging on the human ovarian microenvironment. We obtained donor samples from ovaries at three representative stages of aging: adolescence (~12 years), middle age (~32 years), and old age (~42 years). According to these data, we revealed the transcriptional profiles and spatial gene expression patterns of the EDC, theca cell (TC), and GC in their native microenvironment, as well as the major stromal and immune cell types in the ovary. Notably, we found that with aging, the secretion of DLK1 from TCs increased significantly, and its interaction with the NOTCH protein in *CLDN5* ^+^ blood EDCs intensified, acting as a key factor in the ovarian aging process. Our findings provide vital insights for developing pharmacological approaches to extend the reproductive lifespan in women.

## 2 Results

### 2.1 Overall Design of This Study

**FIGURE 1:**
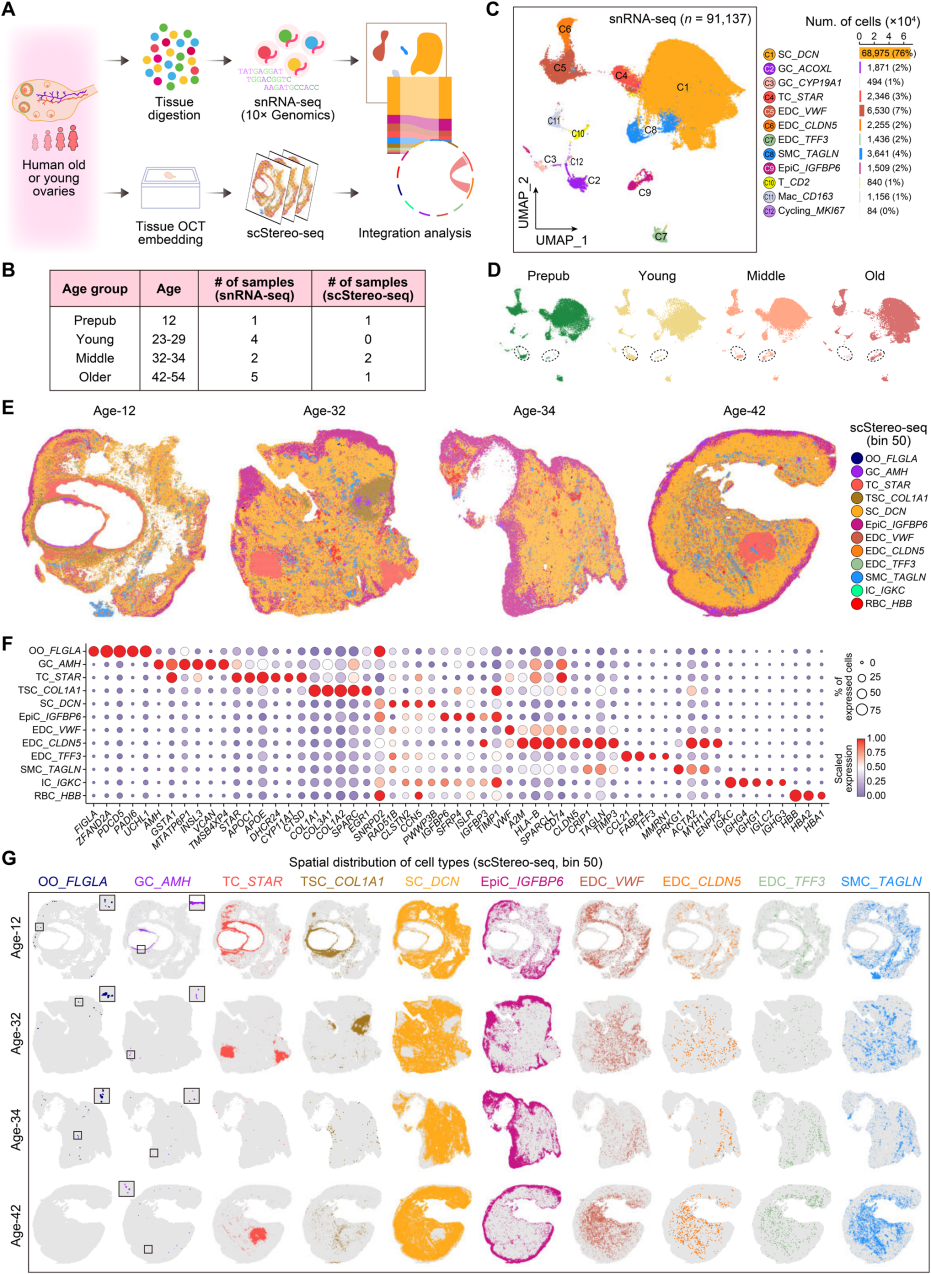
Single‐cell spatiotemporal transcriptomic atlas of the aged human ovary. (A) Schema showing the overall design of this study. (B) Table showing the sample information for snRNA‐seq and scStereo‐seq in this study. (C) Left, UMAP plot showing ovarian cell types revealed by snRNA‐seq data. Right, bar plot showing the cell number of ovarian cell types. The percentage of diverse cell types are indicated in brackets. (D) UMAP plots showing the age information, corresponding to Figure 1C. EpiCs and *ACOXL* ^+^ GCs are indicated with the dashed circle. (E) Spatial visualizations showing the spatial distribution of cell types at ages 12, 32, 34, and 42. Bin 50 is applied in scStereo‐seq data. (F) Dot plot showing the expression profiles of cell‐type‐specific marker genes among scStereo‐seq cell types. Dot size indicates the percentage of expressed spots and dot color indicates the scaled expression level. For each gene, the expression level is scaled among cell types. (G) Separated spatial visualizations showing the spatial distribution of cell types at ages 12, 32, 34, and 42. Some fewer cell types are zoomed in.

The optimal functionality of ovarian activity is generally observed between the ages of 20 and 30, followed by a gradual decline after the age of 30, ultimately leading to the cessation of function around the age of 50 (Secomandi et al. 2022; Dong et al. 2023). To explore the dynamic molecular characteristics of human ovaries across their developmental stages, our study collected healthy ovarian tissues from individuals aged 12, 32, 34, and 42 (Figure 1A,B and Figure S1A). These ovarian tissues were obtained from breast cancer patients who visited the hospital for ovarian tissue cryopreservation to preserve their fertility. Following thorough notification, communication, and consultation, each patient consented to donate a small portion of their ovarian tissue for scientific research purposes. We performed snRNA‐seq on these four ovarian samples using the 10× Genomics platform (Figure 1A,B).

Additionally, to further elucidate ovarian aging dynamics, we integrated snRNA‐seq data from the previous study involving 8 human healthy ovaries (ages 23–54) (Jin et al. 2024), enabling comparative analysis with reproductive prime ovarian samples (Figure 1B). The donors associated with the publicly available data experienced sudden death or bilateral salpingo‐oophorectomy (Jin et al. 2024). All ovarian samples were categorized into four distinct age groups: prepubertal (age 12), young (ages 23–29; mean age 27 years), middle‐aged (ages 32–34; mean age 33 years), and older‐aged (ages 42–54; mean age 50 years) (Figure 1B; Table S1). These samples collectively represent the full spectrum of ovarian development, from immaturity to maturity and eventually aging.

### 2.2 The snRNA‐Seq Atlas of the Human Ovary

After stringent quality control of snRNA‐seq data with the consistent standard, a total of 91,137 single nuclei from 12 human ovaries were retained for subsequent analysis, including 57,970 single nuclei provided by our in‐house data (Figure 1C and Figure S1B). After individual heterogeneity correction, dimension reduction using uniform manifold approximation and projection (UMAP), and graph‐based clustering, we identified 12 cell types from these 12 human ovaries (Figure 1C and Figure S1C). For these retained high‐quality single nuclei, the average number of detected genes and unique molecular identifiers (UMIs) were 1783 and 3304, respectively, and the average mitochondrial RNA contamination percentage was 0.93% (Figure S1B). These cell types were annotated based on the expression profiles of a series of well‐known marker genes, including the SC (*DCN*, *CLDN1*, and *FYB2*), TC [steroidogenic acute regulatory protein (*STAR*), *GSTA1*, and *CYP17A1*], GC (*CYP19A1*, *INHA*, and *AMH*), EDC (*VWF*, *CLDN5*, and *TFF3*), smooth muscle cell (SMC; *RGS5* and *TAGLN*), epithelial cell (EpiC; *CDH11*, *CLDN1*, and *PAX8*), T cell (T; *CD2*), macrophage (Mac; *CD163*), and cycling cell (*MKI67* and *TOP2A*) (Figure 1C). As expected, SC emerged as the predominant cell type within the ovaries, accounting for 76% of the total single‐nuclear population (Figure 1C).

To validate our annotations, we examined the expression profiles of previously established gene signatures (Fan et al. 2019), and observed a high degree of consistency between our annotated cell types and those reported in the previous study (Figure S1D). Additionally, we projected the previous cellular annotations onto our UMAP visualization (Jin et al. 2024), which confirmed the alignment of the annotations (Figure S1E). Moreover, we identified cell‐type‐specific marker genes across these cell types, and their expression patterns further corroborated the accuracy of our cellular annotations (Figure S1F; Table S2). Our analysis revealed that most cell types were present across all age groups, but GCs and EpiCs displayed distinct age‐specific distribution patterns (Figure 1D). Specifically, GCs, especially *ACOXL* ^+^ GCs, were predominantly observed in the prepubertal and young ovaries but were largely absent in the middle‐ and older‐aged ovaries (Figure 1D, indicated by dotted circles). Conversely, EpiCs were primarily captured in middle‐ and older‐aged groups, with minimal presence in the prepubertal and young ovaries.

Of note, no OOs were detected in the snRNA‐seq dataset. To determine whether OOs were captured, we developed a cell scoring model based on the machine learning method using a non‐human primate (NHP) ovarian scRNA‐seq dataset, which included EDCs, GCs, Macs, OOs, SMCs, and SCs (Wang et al. 2020) (see Section 4 for details). The model achieved a prediction accuracy of 96.5% for the NHP dataset and 84.7% for the human dataset (Figure S1G). Notably, no human ovarian cells were classified as OOs, confirming the absence of OOs in the snRNA‐seq dataset (Figure S1G).

Additionally, to further elucidate the transcriptomic conservation of ovarian cell types across species, we collected scRNA‐seq data from mouse ovaries (young: 3 months, old: 9 months) obtained from a previous study (Isola et al. 2024). Using a multi‐class classification approach, we established cross‐species cell type correspondences between human and mouse ovarian cells, as previously described (Peng et al. 2019; Shekhar et al. 2016) (see Section 4 for details). We found that 73% of young mouse ovarian cells and 74% of old mouse ovarian cells matched their human counterparts (Figure S1H). The degree of correspondence, quantified by Adjusted Rand Index, was 0.44 for young human versus young mouse, and 0.59 for old human versus old mouse. These results highlighted observed species‐specific transcriptomic differences, suggesting that 3‐month‐old mouse ovaries were less comparable to ~27‐year‐old human ovaries, whereas 9‐month‐old mouse ovaries showed greater similarity to ~50‐year‐old human ovaries.

### 2.3 The Spatiotemporal Transcriptomic Atlas of the Human Ovary

To decode the spatial aging event of the human ovary at single‐cell resolution, we performed spatial transcriptomic analysis of ages 12, 32, 34, and 42 human ovary tissues using the scStereo‐seq technique (Figure 1A). To achieve an optimal balance of single‐cell resolution and the number of genes captured, we employed a bin size of 50 (50 × 50 bins) for the analysis of the ovarian tissue slice, defining each analyzed unit as a “spot” (Chen et al. 2022). For each spot, the average number of detected genes and transcripts was 705 and 1536, respectively (Figure S2A). By mapping the snRNA‐seq data onto scStereo‐seq slices, we identified 12 distinct ovarian cell types and their corresponding cell‐type‐specific marker genes, followed by Gene Ontology (GO) analysis to elucidate their functional roles (Figure 1E,F and Figure S2B; Table S2). Beyond the cell types revealed by snRNA‐seq, scStereo‐seq detected additional ovarian cell types based on a series of well‐known marker genes. These included the OO expressing *FIGLA*, *PADI6*, *ZP2*, and *ZP4*, which were associated with female gamete generation; theca or stromal cell (TSC) expressing *COL1A1*, *SPARC*, and *EGR1*, which were involved in actin filament organization; IC expressing *IGKC*, *IGHG1*, and *IGLC2*, which was linked to immunoglobulin‐mediated immune response; and red blood cell (RBC) expressing *HBB*, *HBA1*, and *HBA2* (Figure 1E,F; Figures S2B and S3A). Consistent with the snRNA‐seq findings, the scStereo‐seq data also identified SC as the predominant cell type in human ovaries, representing 55% of the total spots (Figure S3B).

We further developed a cell scoring model using a scRNA‐seq dataset of NHP OOs (Wang et al. 2020). This model effectively distinguished four OO subtypes in the publicly available scRNA‐seq dataset (Wang et al. 2020), each corresponding to distinct morphological follicle stages (Figure S3C). Specifically, the model classified 96.4% of human OOs from this study as originating from primordial follicles and 3.6% from primary follicles (Figure S3C). We noticed that OOs were only identified in scStereo‐seq data from the prepubertal ovary; to verify the presence of follicles in the samples used for scStereo‐seq, we performed immunofluorescence staining for STAR and observed the typical concentric layer structure—a hallmark of follicles—across samples from different age groups (Figure S2C). These findings confirm that follicles were indeed present in the original samples. However, the corresponding tissue slices selected for scStereo‐seq failed to capture these structures, suggesting an issue with sample preparation or sectioning rather than an absence of follicles in the donors.

Visualization of the spatial distribution of cell types within the human ovary revealed a well‐organized and structured pattern (Figure 1G and Figure S3D). For instance, ovarian follicles were surrounded by GCs, which were further enclosed by TCs, while the ovarian tissue was predominantly composed of SCs. EpiCs were primarily located at the ovarian periphery, whereas SCs were predominantly concentrated in the central region of the ovary. Additionally, we observed distinct spatial distributions among the three EDC subtypes: *VWF* ^+^ and *CLDN5* ^+^ EDCs exhibited a relatively clustered distribution, while *TFF3* ^+^ EDCs displayed a more scattered pattern.

We also identified a cell population in human ovaries that exhibited high *STAR* expression. Intriguingly, these *STAR* ^+^ cells formed distinct clumps rather than the typical concentric layer structure of TCs, and immunofluorescence staining further confirmed the presence of clustered *STAR* ^+^ cells (Figure 1G and Figure S2D). Of note, the abundance of these clumped *STAR* ^+^ cells increased with ovarian aging, suggesting their progressive accumulation during the aging process (Figure S2E). To determine whether these clumped *STAR* ^+^ cells represent SCs or other cell types, we examined the expression of *PDGFRA*, a classical SC marker gene, in *STAR* ^+^ cell clumps from our scStereo‐seq data (particularly in samples from age 32 to 42 donors). Our analysis revealed a lower *PDGFRA* expression in these *STAR* ^+^ cells compared to *COL1A1* ^+^ TSCs and *DCN* ^+^ SCs, suggesting these clumps are unlikely to be typical SCs (Figure S2F,G). We further performed a systematic comparison of ovarian cell‐type‐specific gene signatures derived from our snRNA‐seq data (Figure S1F; Table S2) with the expression profiles of cell types identified in scStereo‐seq data. This analysis demonstrated that the *STAR* ^+^ cell clumps exhibited a strong expression of established TC marker genes while showing minimal expression of SC marker genes, further supporting their identity as TCs (Figure S2H, blue box). We also observed remarkable consistency between the abundance of TCs identified in snRNA‐seq data (3%) and the proportion of *STAR* ^+^ cell clumps in scStereo‐seq data (5%), providing additional evidence for their shared cellular identity (Figure 1C and Figure S3B). While we determined that the majority of *STAR* ^+^ cell clumps in our scStereo‐seq data represented TCs, our immunofluorescence staining revealed the presence of occasional small‐sized STAR^+^PDGFRA^+^ SCs that did not form large clumps (Figure S2I). Given the critical role of the STAR protein in regulating steroid biosynthesis (Manna et al. 2016), these STAR^+^PDGFRA^+^ SCs may represent steroidogenic SCs that were not captured by the scStereo‐seq method. This observation suggested that although STAR expression can occur in both cell types, their spatial organization and molecular profiles clearly distinguish the predominant TC population from the rare SC population. In our study, clumped STAR^+^ TCs were primarily observed in relatively older donors based on a limited sample size. These structures may arise from alterations in molecular and morphological characteristics. Further investigations using a larger collection of human ovarian samples are warranted to determine whether the presence of clumped STAR^+^ TCs indicates impaired follicular function.

**FIGURE 2:**
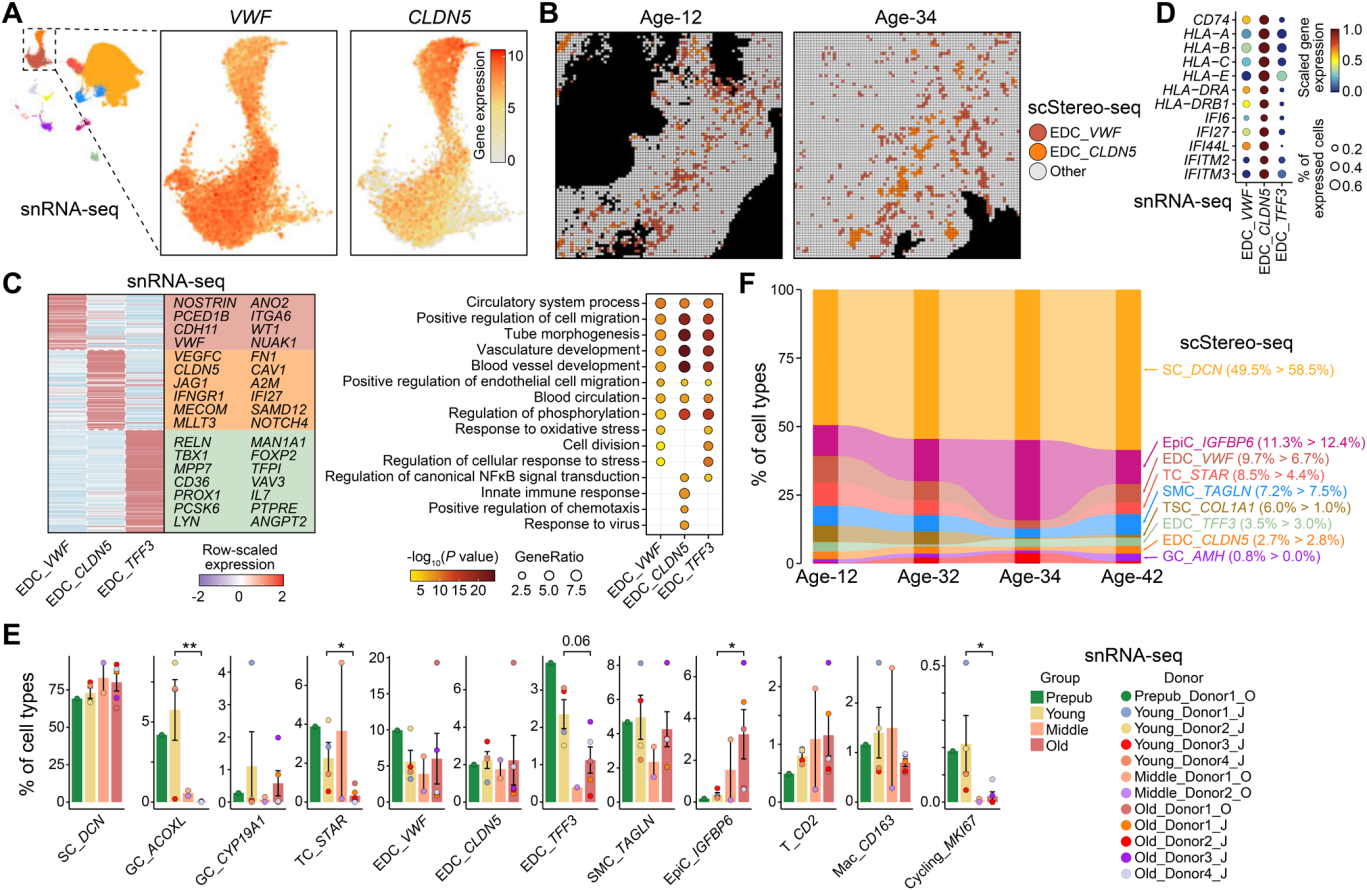
The antigen‐presenting function of *CLDN5* ^+^ blood EDCs in the human ovarian microenvironment. (A) UMAP plots showing the expression level of *VWF* and *CLDN5* among blood EDCs. (B) Spatial visualizations showing the spatial distribution of *VWF* ^+^ and *CLDN5* ^+^ blood EDCs at ages 12 and 34. (C) Left, heatmap showing the row‐scaled expression level of cell‐type‐specific marker genes among diverse EDC subtypes. Representative genes are listed at right. Right, dot plot showing GO terms corresponding to cell‐type‐specific marker genes of EDC subtypes. Sizes and colors indicate GeneRatio and significant statistic of GO terms, respectively. (D) Dot plot showing the expression level of cell‐type‐specific marker genes of *CLDN5* ^+^ blood EDCs. Sizes and colors indicate the percentage of expressed cells and row‐scaled expression level by snRNA‐seq data, respectively. (E) Bar plots showing the percentage of cell types among all snRNA‐seq cells. Bar and dot colors indicate the age group and donor information, respectively. One‐tailed Wilcoxon‐ranked sum test is performed between the older‐aged and young groups, and *p* values with statistical significances are indicated, in which *p < 0.05 and **p < 0.01. Data are shown as mean ± SEM. (F) Sankey plot showing the altered composition of distinct cell types at each age. The percentage corresponding to ages 12 and 42 is listed in brackets, respectively.

In summary, we described a comprehensive aging atlas across distinct regions of the human ovary using single‐cell spatial transcriptomic profiling.

### 2.4 The Antigen‐Presenting Function of *CLDN5* ^+^ Blood EDCs in Human Ovarian Microenvironment

The previous study has identified two EDC subtypes: blood EDCs characterized by high expression of *VWF* and *FLT1*, and lymphatic EDCs marked by elevated expression of *FLT4* and *PROX1* (Jin et al. 2024). In our analysis, we further refined the classification of blood EDCs into two distinct subtypes: *VWF* ^high^ *CLDN5* ^low^ and *VWF* ^low^ *CLDN5* ^high^ blood EDCs, and we defined them as *VWF* ^+^ and *CLDN5* ^+^ blood EDCs hereafter, respectively (Figure 2A). These subtypes exhibited a distinct spatial distribution within the scStereo‐seq slices of human ovaries (Figure 2B).

To elucidate the molecular characteristics of these EDC subtypes, we performed DEG analysis on snRNA‐seq data to identify cell‐type‐specific marker genes, which revealed distinct expression profiles across three EDC subtypes (Figure 2C; Table S2). GO analysis showed that the marker genes of three EDC subtypes were all involved in vasculature development (i.e., *CD34*, *CDH5*, and *CDH13*) and positive regulation of cell migration (i.e., *RHOA*, *EGFR*, and *VEGFC*) (Figure 2C). The marker genes of *TFF3* ^+^ EDCs, including *FLT4* and *PROX1*, were involved in regulating cellular response to stress (i.e., *KLF4*, *DDX5*, and *ACTB*) and modulating canonical NFκB signaling transduction (i.e., *RHOA*, *GJA1*, and *STAT1*), further supporting their classification as lymphatic EDCs (Figure 2C). In addition to the role in blood vessel development (i.e., *CDH5*, *NOTCH4*, and *VEGFC*) and the regulation of canonical NFκB signaling transduction (i.e., *BST2*, *CAV1*, and *IKBKB*), *CLDN5* ^+^ blood EDCs also exhibited upregulation of genes associated with the innate immune response (i.e., *ACTG1*, *B2M*, *IFNAR2*, and *IFNGR1*) and positive regulation of chemotaxis (i.e., *CD74*, *CXCL12*, and *FGF2*) (Figure 2C). Notably, *CLDN5* ^+^ blood EDCs displayed specific high expression of Major Histocompatibility Complex (MHC)‐related genes, such as *HLA‐A*, *HLA‐B*, and *HLA‐DRA*, along with interferon (IFN)‐stimulated genes (ISGs) and IFN receptors, including *IFI6*, *IFITM2*, *IFNAR2*, and *IFNGR1* (Figure 2D). Emerging evidence highlights the existence of “immunomodulatory EDCs” across diverse tissues—including liver sinusoidal EDCs—that extend beyond their classical roles in alloimmunity, immune cell recruitment, immune tolerance, and vascular inflammation (Sørensen et al. 2012; Stamatiades et al. 2016; Amersfoort et al. 2022; Pober and Sessa 2007). Notably, specific EDC subtypes function as semiprofessional antigen‐presenting cells (APCs), bridging innate and adaptive immunity (Amersfoort et al. 2022). In line with this, our results identified *CLDN5* ^+^ blood EDCs as semiprofessional APCs to modulate immune responses within the ovarian microenvironment.

### 2.5 Age‐Related Variations in the Proportion of Ovarian Cell Types

We next investigated the temporal alterations in cellular composition within the human ovary across various age groups. SCs, the predominant cell type in the ovary, exhibited an upward trend in abundance with ovarian aging (Spearman's correlation coefficient *ρ* = 0.5, *p* = 0.1), a pattern corroborated by both snRNA‐seq and scStereo‐seq datasets (Figure 2E,F). EpiCs, which form the ovarian surface, are essential for post‐ovulatory repair and undergo cyclic expansion and contraction in synchrony with ovarian changes (Hartanti et al. 2019). Our analysis revealed a significant age‐related increase in the proportion of EpiCs (*ρ* = 0.5, *p* = 0.07; one‐tailed Wilcoxon‐ranked sum test *p* = 0.03 between older‐aged and young groups), suggesting an expansion of EpiCs in the older‐aged group.

Conversely, the abundances of *ACOXL* ^+^ GCs (*ρ* = −0.9, *p* = 0.006; Wilcoxon *p* = 0.008) and TCs (*ρ* = −0.8, *p* = 0.003; Wilcoxon *p* = 0.02) exhibited a significant decline with advancing age, potentially reflecting the reduction in follicle numbers (Figure 2E,F). Additionally, lymphatic EDCs demonstrated a decrease in abundance during aging, decreasing from 2.4% in the young group to 1.1% in the older‐aged group according to snRNA‐seq data (Wilcoxon *p* = 0.06), or from 3.5% at age 12 to 3.0% at age 42 according to scStereo‐seq data. In contrast, blood EDCs maintained a relatively stable abundance across age groups. Furthermore, as anticipated, the proportion of cycling cells was markedly reduced in the older‐aged group (*ρ* = −0.6, *p* = 0.02; Wilcoxon *p* = 0.02).

### 2.6 Mitochondrional Dysfunction, Loss of Cell Identity, and Enhanced *CLDN5* ^+^ Blood EDC Activity in Aged Human Ovaries

**FIGURE 3:**
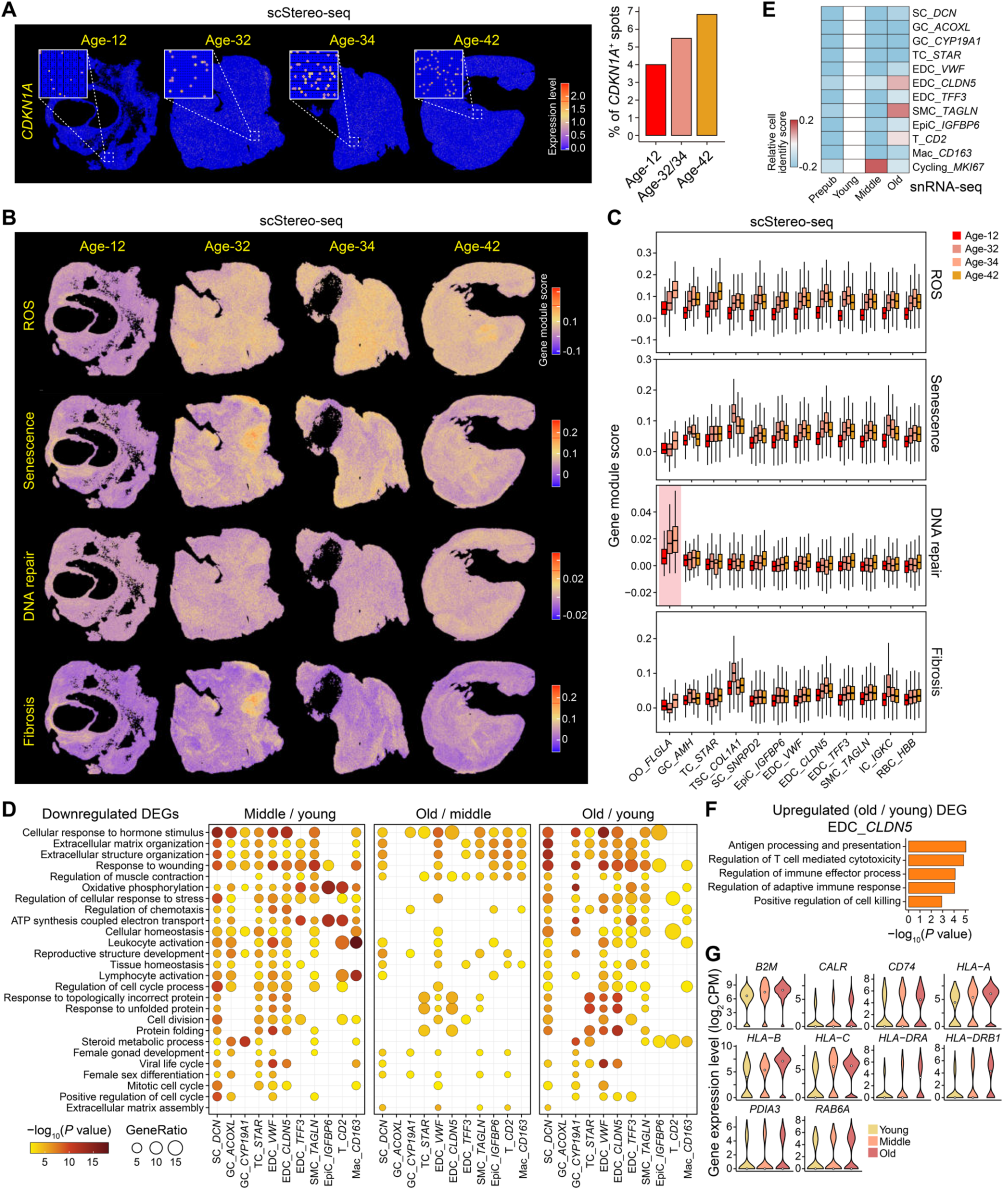
Dynamic expression profiling of aging hallmarks during ovarian aging. (A) Left, spatial visualizations showing the expression level of *CDKN1A*. Representative regions are zoomed in. Right, bar plot showing the percentage of *CDKN1A* ^+^ spots among all ovarian spots at diverse ages. (B) Spatial visualizations showing the gene module score of representative aging hallmarks. (C) Boxplots showing the gene module score of representative aging hallmarks. OOs are highlighted. Corresponding to Figure 3B. (D) Dot plots showing GO terms corresponding to the aging‐related downregulated DEGs between middle‐aged and young groups (left), older‐ and middle‐aged groups (middle), or older‐aged and young groups (right). Sizes and colors indicate GeneRatio and significant statistic of GO terms, respectively. (E) Heatmap showing the cell identity of diverse age groups relative to the young group, with red and blue colors representing the acquisition or loss of cell identity, respectively. (F) Bar plot showing GO terms corresponding to aging‐related upregulated DEGs between older‐aged and young groups in *CLDN5* ^+^ blood EDCs. (G) Violin plots showing the expression levels of antigen processing and presentation‐related genes in *CLDN5* ^+^ blood EDCs from diverse age groups. These genes significantly upregulated in the older‐aged group compared to the young group. The white dot indicates the median value.

To explore whether cellular senescence burden increased during human ovarian aging, we examined the prevalence of *CDKN1A* (*p21*)‐positive and *CDKN2A* (*p16*)‐positive spots within spatial slices. While the expression of *CDKN2A* was detected in only a limited number of spots, a higher quantity of *CDKN2A* ^+^ spots was observed at age 42 compared to age 12 (Figure S4A). In alignment with this finding, we detected a progressive increase in the abundance of *CDKN1A* ^+^ spots with advancing age (Figure 3A). Moreover, we examined the expression level of classic aging hallmarks (Bao et al. 2023). As expected, reactive oxygen species (ROS)‐, senescence‐, DNA repair‐, and fibrosis‐related genes were markedly upregulated in aged human ovaries compared with the prepubertal ovary (Figure 3B,C and Figure S4B). Furthermore, when analyzing distinct ovarian cell types, we observed that DNA repair‐related genes were expressed at relatively higher levels in OOs compared to somatic cell types, likely due to the active meiosis and mitosis in OOs (Figure 3C). These genes exhibited lower expression in the prepubertal ovary compared to other age groups, consistent with the quiescent state of OOs during this developmental stage.

To further elucidate transcriptomic changes associated with aging, we identified aging‐related DEGs between adjacent age stages using snRNA‐seq data (Table S3). We found that aging‐related downregulated DEGs were enriched in extracellular matrix organization (i.e., *COL1A1*, *COL1A2*, and *TGFB1*), oxidative phosphorylation (i.e., *ND1*, *UQCRQ*, *COX7A2*, and *ATP5PF*), cellular homeostasis (i.e., *ANXA6*, *APOE*, *GATA4*, and *SMAD2*), and cell cycling (i.e., *APP*, *CDK9*, and *UBE2S*) (Figure 3D). Genes encoding mitochondrial complex I~V (excluding complex II) were downregulated in the older‐aged group, accompanied by an increased expression of ROS‐related genes, suggesting impaired mitochondrial function and ROS accumulation during ovarian aging (Figure 3B,D). Focusing on the antioxidant genes collected from the previous study (Ren et al. 2021), we observed that these genes exhibited significantly higher expression levels in ovarian cells from young individuals compared to middle‐ and older‐aged groups (Figure S4C). This age‐related decline in antioxidant gene expression was consistently observed across multiple cell types. For example, TCs demonstrated a downregulation of antioxidant gene expression in the older individuals relative to the young counterpart, a finding that corroborates our earlier observations (Wang et al. 2020). Additionally, aging‐related downregulated DEGs were linked to reproductive structure development (i.e., *FOXO3*, *GATA6*, and *SOD1*), female gonad development (i.e., *CYP19A1*, *IDH1*, *INHA*, and *UBB*), and female sex differentiation (i.e., *INHBB*, *CSMD1*, and *DACH2*), reflecting functional decline in aged ovaries, as expected (Figure 3D). Conversely, aging‐related upregulated DEGs were enriched in the apoptotic signaling pathway (i.e., *BCL2* and *BNIP3*), regulation of the Target of Rapamycin (TOR) signaling (i.e., *HIF1A*, *SAMTOR*, and *RICTOR*), cellular senescence (i.e., *SMC5*, *MAPK14*, and *IGF1R*), and antigen processing and presentation (i.e., *CD74*, *HLA‐A*, and *HLA‐B*) (Figure S4D). Interestingly, it is reported that inhibition of mechanistic TOR (mTOR) can delay ovarian aging in mice (Heng et al. 2021).

To explore the molecular links between ovarian aging and reproductive disorders, we examined the overlap between DEGs in older‐aged versus young ovaries and a curated gene set associated with ovarian diseases, including premature ovarian failure (POF) and primary ovarian insufficiency (POI) (Wang et al. 2020). Our analysis revealed that *CYP19A1* ^+^ GCs exhibited significant downregulation of multiple POF/POI‐related genes, including known candidates such as *FSHR*, *FOXL2*, and *NR5A1* (Jiao et al. 2018) (Figure S4E,F). Additionally, we observed age‐related downregulation of other steroidogenic genes, such as *CYP11A1*, in *CYP19A1* ^+^ GCs (Figure S4F). Notably, *ZP2*, which promotes OO development, was significantly downregulated at ages 32/34 compared to age 12, while the expression levels of other OO development‐related genes (i.e., *ZP1*, *FST*, and *GDF9*) remained stable across age groups (Figure S4F). These findings suggested that age‐associated transcriptional changes in GCs may contribute to the molecular underpinnings of POF/POI and ovarian dysfunction.

Furthermore, we compared the transcriptomic profiles of young and prepubertal ovaries. Our findings indicated that ovarian functions, which declined during aging, were established from prepuberty to ovarian maturity (Figure S4G). These functions include cellular response to hormone stimulus, oxidative phosphorylation, and cell cycle regulation.

We next explored whether cell identity underwent heterogeneous alterations during ovarian aging (see Section 4 for details). Briefly, using snRNA‐seq data, we first conducted DEG analysis across cell types to identify cell‐type‐specific marker genes within cells from the young ovary, which we defined as the “cell identity gene”. We then calculated the gene module score of these cell identity genes in the corresponding cell types from each age group, termed the “cell identity score”. Compared to the young ovary, the prepubertal ovary showed a reduced cell identity score, indicative of its immature state and function (Figure 3E). Of note, the cell identity score decreased in most cell types within the middle‐ and older‐aged groups, reflecting a loss of cell identity during ovarian aging (Figure 3E). Unexpectedly, *CLDN5* ^+^ blood EDCs and SMCs exhibited increased cell identity scores in the older‐aged group compared to the young group. To further investigate, we performed GO analysis on aging‐related upregulated DEGs in *CLDN5* ^+^ blood EDCs. The enriched GO terms included antigen processing and presentation, as well as regulation of immune effector process, aligning with the enhanced cell identity score observed in these cells, as semiprofessional APCs, in the older‐aged group (Figure 3F). When observing the expression levels of antigen processing and presentation‐related upregulated DEGs, we found that these genes were significantly upregulated in the older‐aged group compared to the young and middle‐aged groups (Figure 3G). These findings suggested that *CLDN5* ^+^ blood EDCs, as semiprofessional APCs, played an enhanced role in modulating immune responses within the aged human ovarian microenvironment.

### 2.7 Activated Inflammatory Microenvironment in Aged Human Ovaries

**FIGURE 4:**
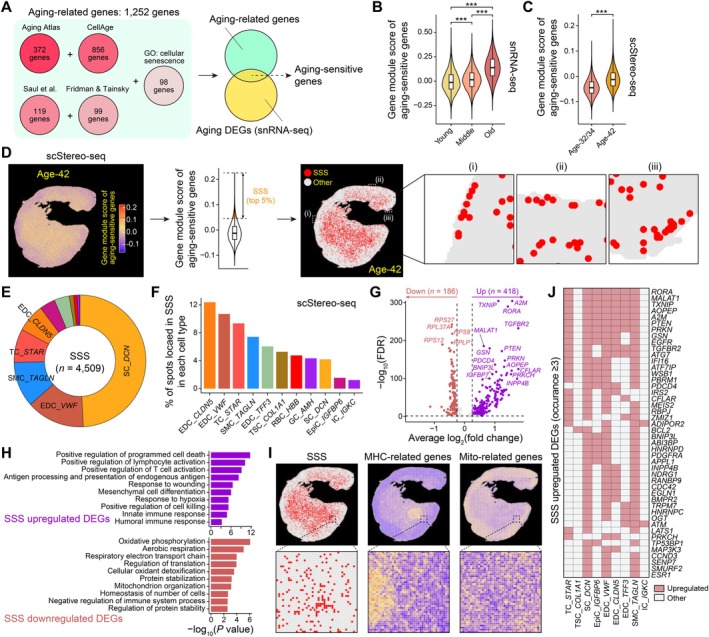
Activated inflammatory microenvironment in aged human ovaries. (A) Schema showing the process to obtain aging‐sensitive genes. (B) Violin plot showing the gene module score of aging‐sensitive genes in diverse age groups, according to the snRNA‐seq dataset. Two‐tailed Student's *t*‐test *p* values are indicated, in which ***p < 0.001. (C) Violin plot showing the gene module score of aging‐sensitive genes in diverse ages, according to the scStereo‐seq dataset. Two‐tailed Student's *t*‐test *p* value is indicated, in which ***p < 0.001. (D) Left, spatial visualization showing the gene module score of aging‐sensitive genes at age 42. Middle, violin plot showing the gene module score of aging‐sensitive genes at age 42, according to the scStereo‐seq dataset. The top‐ranked 5% of spots with the highest gene module scores of aging‐sensitive genes were defined as SSSs. Right, spatial visualization showing the distribution of SSSs at age 42. Three marginal regions with SSSs are zoomed in. (E) Pie chart showing the percentage of diverse cell types corresponding to SSSs. These spots locate in SSSs. (F) Bar plot showing the percentage of spots located in SSSs within each cell type. (G) Volcano plot showing the DEGs between SSSs and other spots (without cell type separation). The numbers of upregulated and downregulated DEGs are indicated in brackets. Representative DEGs are indicated. (H) Bar plots showing GO terms corresponding to upregulated (top) and downregulated (bottom) DEGs between SSSs and other spots (without cell type separation). (I) Left, spatial visualization showing the distribution of SSSs at age 42. Middle and right, spatial visualizations showing the gene module scores of MHC‐ (*HLA‐A*, *HLA‐B*, *HLA‐C*, *HLA‐E*, *HLA‐DRA*, and *HLA‐DRB1*) and mitochondrial‐related genes (*ATP5PO*, *ATP5MG*, *COX4I1*, *COX6C*, *COX7C*, *ND4L*, *UQCRB*, and *UQCR10*) at age 42. Representative regions are zoomed in. (J) Heatmap showing the distribution of upregulated DEGs between SSSs and other spots within diverse cell types. Only upregulated DEGs occur in at least three cell types are shown.

To deeply characterize the ovarian microenvironment within senescence hotspots, we identified “senescence sensitive spots (SSSs)” as previously reported (Ma et al. 2024). Briefly, we integrated five well‐defined senescence gene sets from prior studies (Avelar et al. 2020; Fridman and Tainsky 2008; Aging Atlas Consortium 2021; Saul et al. 2022), and intersected these genes with our aging‐related upregulated DEGs to identify “aging‐sensitive genes” (Figure 4A and Figure S5A; Table S4). Utilizing snRNA‐seq data, we calculated the gene module scores of these aging‐sensitive genes across single nuclei from different age groups. We observed a significant and gradual upregulation of these genes from the young to middle‐ and older‐aged groups, which validated the reliability of the identified aging‐sensitive genes (Figure 4B). Additionally, we detected a notable upregulation of these genes in scStereo‐seq spots at age 42 compared to ages 32/34 (Figure 4C).

For the ovary from age 42, we defined the top‐ranked 5% of spots with the highest gene module scores of aging‐sensitive genes as SSSs (*n* = 4509) (Figure 4D, left and middle). Most SSSs were concentrated in the central region, though a few were also detected in the peripheral region, indicating that aging events occurred in both the ovarian medulla and surface (Figure 4D, right). SSSs were identified across various cell types, with 49% overlapping with SCs (Figure 4E). Notably, *CLDN5* ^+^ and *VWF* ^+^ blood EDCs exhibited the highest abundance SSSs (12% and 11%, respectively), highlighting their heightened sensitivity to aging (Figure 4F).

Further analysis revealed 418 upregulated and 186 downregulated DEGs between SSSs and non‐SSSs using scStereo‐seq data (Figure 4G; Table S4). Among the upregulated DEGs were several MHC‐ (i.e., *CD74*, *HLA‐B*, and *HLA‐DRA*) and complement‐related genes (i.e., *C3* and *C7*). GO results showed that SSS‐upregulated DEGs were enriched in antigen processing and presentation (i.e., *CD74*, *HLA‐B*, and *HLA‐DRA*), positive regulation of lymphocyte activation (i.e., *IL6ST* and *STAT5B*), innate immune response (i.e., *C3*, *IFI16*, *JAK1*, and *RAF1*), humoral immune response (i.e., *B2M* and *CXCL12*), response to hypoxia (i.e., *ATM*, *MALAT1*, and *RORA*), and positive regulation of programmed cell death (i.e., *BCL2*, *BCL6*, and *ABL1*), reflecting an enhanced inflammatory microenvironment (Figure 4H). In contrast, SSS‐downregulated DEGs were associated with oxidative phosphorylation (i.e., *COX6C* and *ATP5PO*) and homeostasis of cell number (i.e., *HMOX1* and *PRDX2*). Spatial expression analysis revealed a mutually exclusive pattern: MHC‐related genes were highly expressed in the central region, while oxidative phosphorylation‐related genes were more abundant in the peripheral region (Figure 4I).

We also identified transcriptomic differences between SSSs and non‐SSSs across various cell types (Figure 4J; Table S4). Interestingly, several commonly upregulated genes were observed across cell types, including *PTEN*, *TGFBR2*, *ATG7*, and *ESR1*, which are implicated in primary ovarian insufficiency (Figure S5B). Additionally, other ovarian function‐related genes were upregulated in SSSs, such as those involved in female gonad development (i.e., *A2M* and *PDGFRA*) and the ovulation cycle (i.e., *EGFR* and *ESR1*) (Figure S5B). These findings suggest that SSSs serve as epicenters for heightened inflammation, which may compromise the microenvironment essential for maintaining core ovarian functions.

### 2.8 The Accumulation of Immunoglobulin‐Expressing Cells During Ovarian Aging

**FIGURE 5:**
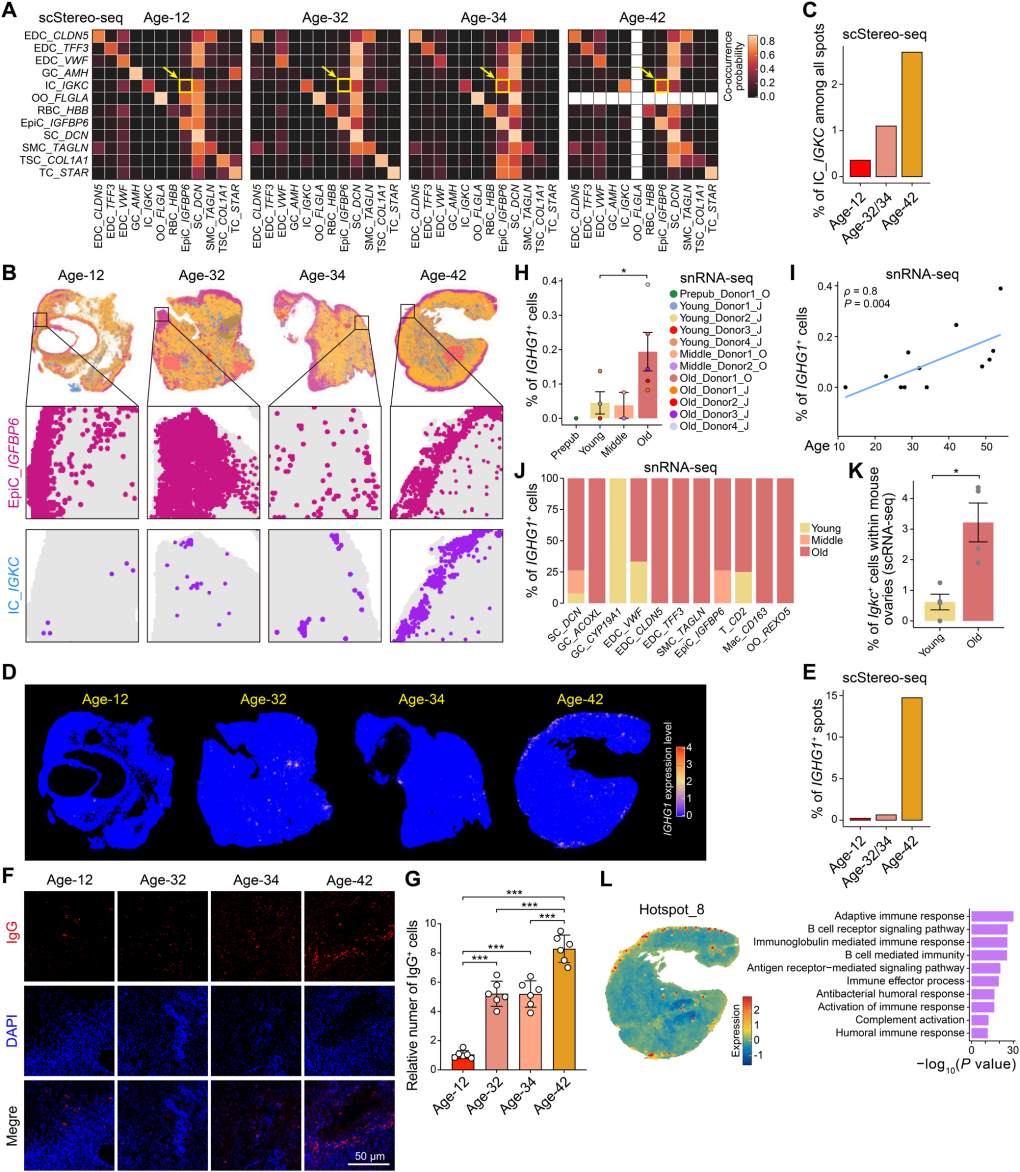
The accumulation of immunoglobulin‐expressing cells during ovarian aging. (A) Heatmaps showing the co‐occurrence probability of any two cell types in spatial distribution at diverse ages. The co‐occurrence of ICs and EpiCs is highlighted with yellow arrows and boxes. (B) Spatial visualizations showing the spatial distribution of EpiCs and ICs at ages 12, 32, 34, and 42. Representative regions are zoomed in. (C) Bar plot showing the percentage of ICs among all ovarian spots at diverse ages. (D) Spatial visualizations showing the expression level of *IGHG1*. (E) Bar plot showing the percentage of *IGHG1* ^+^ spots among all ovarian spots at diverse ages. (F) Immunofluorescence staining of IgG within the human ovary at ages 12, 32, 34, and 42. Scale bar, 50 μm. (G) Bar plot showing the relative number of IgG^+^ cells within the human ovary at age 32, 34, and 42, compared to age 12. Two‐tailed Student's *t*‐test is performed, in which ***p < 0.001. Data are shown as mean ± SEM. (H) Bar plot showing the percentage of *IGHG1* ^+^ cells among all human snRNA‐seq cells used in this study. Bar and dot colors indicate the age group and donor information, respectively. One‐tailed Wilcoxon‐ranked sum test is performed between the older‐aged and young groups, in which *p < 0.05. Data are shown as mean ± SEM. (I) Scatter plot showing the relationship between the percentage of *IGHG1* ^+^ cells among all human snRNA‐seq cells used in this study and women ages. Spearman's correlation coefficient (*ρ*) and correlation test significance (*p*) are indicated. (J) Stacked bar plot showing the percentage of *IGHG1* ^+^ cells in diverse age groups, with cell type separation. (K) Bar plot showing the percentage of *Igkc* ^+^ cells in mouse ovaries. The scRNA‐seq data of mouse ovaries are collected from the previous study (Isola et al. 2024). Dot indicates each mouse ovary. One‐tailed Wilcoxon‐ranked sum test is performed, in which *p < 0.05. Data are shown as mean ± SEM. (L) Left, spatial visualization showing the expression level of spatial hotspot_8. Right, bar plot showing GO terms corresponding to spatial hotspot_8.

To investigate gene modules with genes whose expression varies in a similar way among cells nearby them, we characterized spatial hotspots using our scStereo‐seq data. For each age, we identified 9–11 spatial hotspots and performed GO analysis for these hotspots (Figure S6 and Figure 5L; Table S5). We found that the spatial regions with the highest expression of hotspots were primarily distributed along cell types, supported by GO enriched terms. For example, SMCs were in the spatial region with the highest expression of hotspot_1 at age 12, and these hotspot‐related genes were involved in the muscle system process (Figure S6B). And EpiCs were in the spatial region with the highest expression of hotspot_2 at age 32, and these hotspot‐related genes were enriched in extracellular matrix organization (Figure S6D).

Furthermore, to explore whether the spatial organization changed during ovarian aging, we performed cell co‐occurrence analysis with scStereo‐seq data. The spatial neighbor relationships remained relatively stable for most cell types during aging; for example, SCs consistently exhibited proximity to other cell types (Figure 5A). However, we observed an increased co‐occurrence probability between ICs and EpiCs with advancing age (Figure 5A,B). We also detected a higher proportion of ICs among all ovarian spots in the aged human ovarian microenvironment (Figure 5C). Given that ICs specifically expressed high levels of immunoglobulin‐related genes, such as *IGHG1/3/4* and *IGLC2* (Figure 1F), we further analyzed the proportion of *IGHG1* ^+^ spots and found an increase in their abundance at age 42 compared to other ages (Figure 5D,E). To validate this observation, we performed immunofluorescence staining, which further confirmed the accumulation of IgG^+^ cells during ovarian aging (Figure 5F,G).

Consistent with the increased immunoglobulin infiltration observed in aged ovaries through scStereo‐seq data, snRNA‐seq data demonstrated a significantly higher proportion of *IGHG1* ^+^ cells in the older‐aged group compared to the young group (one‐tailed Wilcoxon‐ranked sum test *p* = 0.03) (Figure 5H). Notably, the proportion of *IGHG1* ^+^ cells in human ovaries showed a positive correlation with women's ages (Spearman's correlation coefficient *ρ* = 0.8, *p* = 0.004) (Figure 5I). Across nearly all cell types (excluding *CYP19A1* ^+^ GCs), *IGHG1* ^+^ cells were predominantly detected in the older‐aged group rather than other age groups (Figure 5J). We also quantified the abundance of *Igkc* ^+^ cells (*Ighg1* was not detected in the mouse dataset) and observed a significant increase in aged mouse ovaries compared to young mice (*p* = 0.01) (Figure 5K). This finding supported the cross‐species conversion of immunoglobulin‐expressing cell accumulation during ovarian aging.

We found that ICs were primarily located in the spatial region with the highest expression of spatial hotspot_8 related genes, and GO analysis demonstrated that these hotspot‐related genes were involved in immunoglobulin‐mediated immune response, antigen receptor‐mediated signaling pathway, and humoral immune response (Figure 5L). Notably, ICs were primarily located at the ovarian edge, like EpiCs, indicating that the occurrence of immunoglobulin‐expressing cells was not random in the ovarian microenvironment (Figures 1D, 5B and Figure S3D).

### 2.9 The Enhanced Interaction Between TCs and *CLDN5* ^+^ Blood EDCs Through the *DLK1:NOTCH3* Axis During Ovarian Aging

**FIGURE 6:**
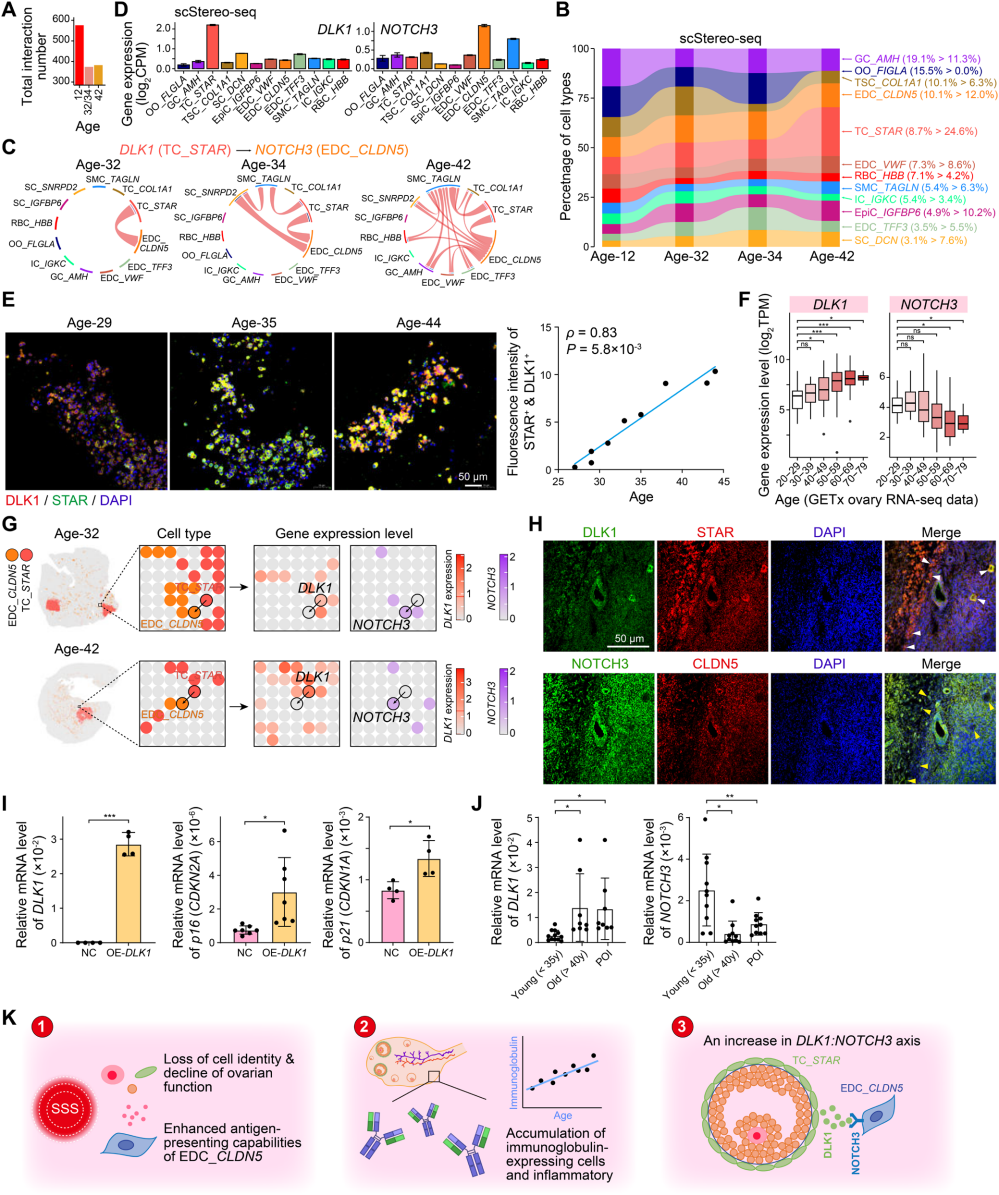
Dynamic cell–cell interactions among ovarian cell types during aging. (A) Bar plot showing the overall number of cell–cell interactions across ages. (B) Sankey plot showing the altered percentage of cellular interactions in distinct cell types at each age. The percentage corresponding to age 12 and 42 is listed in brackets, respectively. (C) Chord plots showing the usage of ligand *DLK1* and its receptor *NOTCH3*. (D) Bar plots showing the expression level of *DLK1* and *NOTCH3* across cell types. Data are shown as mean ± SEM. (E) Left, immunofluorescence staining of DLK1 and STAR in follicular fluid samples from women of varying age. Right, scatter plot showing the correlation of the fluorescence density of DLK1 in STAR^+^ TCs and the age of women. The Spearman's correlation coefficient (ρ) and correlation test value (*p*) are indicated. Scale bar, 50 μm. (F) Boxplots showing the expression level of *DLK1* and *NOTCH3* across ages. These ovarian RNA‐seq data from distinct ages are all obtained from GTEx database. (G) Left, spatial visualizations showing the cell type of *STAR* ^+^ TCs and *CLDN5* ^+^ EDCs. Some regions are enlarged. Right, spatial visualizations showing the expression level of *DLK1* and *NOTCH3*, respectively. The spatial region is as same as the left panel. The scale of color bars is varied among ages and genes. (H) Immunofluorescence staining of DLK1 in STAR^+^ TCs and NOTCH3 in CLDN^+^ blood EDCs in the human ovary. Scale bar, 50 μm. (I) Bar plots showing the RT‐qPCR relative expression level of *DLK1* (left), *p16* (*CDKN2A*) (middle), and *p21* (*CDKN1A*) (right) after overexpressing *DLK1* within the human GC cell line. Data are shown as mean ± SEM. Two‐tailed *t*‐test *p* values are calculated, in which *p < 0.05 and ***p < 0.01. (J) Bar plots showing the relative expression level of *DLK1* (left) and *NOTCH3* (right) using RT‐qPCR. Data are shown as mean ± SEM. Two‐tailed *t*‐test *p* values are calculated, in which *p < 0.05 and **p < 0.01. (K) Cartoon showing the main aging‐related findings revealed by our study.

Altered intercellular communication is a hallmark of aging (López‐Otín et al. 2023). To decipher the dynamic changes of cell–cell interactions during ovarian aging, we conducted ligand‐receptor interaction analysis for each human ovary tissue based on scStereo‐seq data. During ovarian aging, the overall number of cell–cell interactions decreased from 576 interactions in the prepubertal ovary to an average of 376 interactions in the older‐aged ovary, suggesting that the aging process led to a reduction in cellular connectivity (Figure 6A). We found that the major cell–cell interactions were distributed between GCs and OOs in the prepubertal ovary, whereas a relatively higher proportion of interactions emerged in TCs, *CLDN5* ^+^ blood EDCs, EpiCs, and SCs during ovarian aging (Figures 6B and Figure S7A).

Specifically, in the ovary at age 32, we discovered that delta‐like 1 homolog (DLK1), encoded by *DLK1* and expressed by TCs, acted as a ligand on the receptor NOTCH3 expressed by *CLDN5* ^+^ blood EDCs (Figure 6C). However, the *DLK1:NOTCH3* axis was undetectable in any cellular interactions at age 12. With increasing age, the *DLK1:NOTCH3* axis was not only restricted between TCs and *CLDN5* ^+^ blood EDCs but also expanded to involve more cell types (Figure 6C). *DLK1* exhibited a highly specific expression pattern in TCs (Figure 6D and Figure S7B), with its expression level significantly elevating in middle‐ and older‐aged ovaries compared to the prepubertal ovary (Figure S7C). This observation was further validated in an independent scRNA‐seq dataset of human ovaries (Wu et al. 2024), where *DLK1* expression was significantly higher in middle‐ and older‐aged human ovaries than in young ovaries (Figure S7D). In contrast, *NOTCH3* showed a particularly high expression level in *CLDN5* ^+^ blood EDCs (Figure 6D and Figure S7B), and its expression remained relatively stable from age 12 to 42 (Figure S7C).

To further validate our findings, we collected follicular fluid samples from women across a broad age range and subsequently performed immunofluorescence staining to quantify DLK1 protein level in *STAR* ^+^ TCs (Figure 6E). We observed a significantly positive correlation between DLK1 fluorescence intensity and age in TCs (Spearman's correlation coefficient *ρ* = 0.83, *p* = 5.8 × 10^−3^) (Figure 6E). Additionally, we analyzed ovarian RNA‐seq data from the GTEx database, encompassing a wide age range (20–79 years), to assess *DLK1* and *NOTCH3* expression in a larger cohort of 180 human ovarian samples. We found that *DLK1* expression progressively increased from age 20 to 79, while *NOTCH3* expression remained relatively stable from age 20 to 49 and showed slight downregulation after age 50 (Figure 6F). These results aligned with our scStereo‐seq data, strongly supporting the potential of *DLK1* as a diagnostic marker for ovarian aging.

Subsequently, we examined the spatial expression of the interactive signal *DLK1:NOTCH3* and found that the ligand *DLK1* was highly expressed in TCs, and the receptor *NOTCH3* was highly expressed in *CLDN5* ^+^ blood EDCs in close proximity to TCs (Figure 6G). We further verified this proximity of these two cell types using immunofluorescence staining (Figure 6H). This spatial proximity suggested that it facilitates signaling interactions between these two cell types.

We next investigated the functional roles of *DLK1* in the human aging process through in vitro experiments. As established human TC cell lines are not currently available, and obtaining primary human TCs from healthy women remains technically challenging, we utilized the human GC cell line SVOG as an alternative model. Upon overexpression of *DLK1* in SVOG cells at passage 2, we observed a significant upregulation of the aging biomarkers *p16* (*CDKN2A*) (two‐tailed *t*‐test *p* = 0.025) and *p21* (*CDKN1A*) (*p* = 0.016) in the transfected cells (Figure 6I). This result aligned with our bioinformatics analysis, which indicated elevated *DLK1* expression in aging human ovaries.

To further explore the clinical relevance of *DLK1* and *NOTCH3* expression alterations, we investigated their levels in ovarian diseases, specifically POI. Primary GCs were collected from three donor groups during assisted reproductive technology treatment: healthy young women (age < 35 years), healthy older women (age > 40 years), and POI patients (age < 35 years). Quantification of *DLK1* and *NOTCH3* expression revealed that, compared to young women, the *DLK1* level was significantly higher in older women (two‐tailed *t*‐test *p* = 0.046) while the *NOTCH3* level was significantly lower (*p* = 0.011), consistent with our analysis results from the GTEx data (Figure 6J). Importantly, compared to young women, the *DLK1* expression was also significantly elevated in POI patients (*p* = 0.041), and the *NOTCH3* expression was significantly reduced (*p* = 0.008) (Figure 6J).

## 3 Discussion

Ovarian aging is a significant factor affecting fertility, and understanding its mechanisms is essential for the preservation of female fertility. With the advanced single‐cell technology, transcriptome characteristics of aging ovaries have been extensively studied across several species, including humans, NHPs ( *Macaca fascicularis* ), and mice (Wang et al. 2020; Fan et al. 2019; Isola et al. 2024). However, the ovary, as a tissue with a highly heterogeneous architecture, contains not only a variety of complex cell types but also cellular interactions that are dependent on the spatial microenvironment, which has not been fully elucidated before.

Hence, we constructed a comprehensive spatial aging atlas of prepubertal, young, middle‐, and older‐aged human ovaries using snRNA‐seq and the state‐of‐the‐art scStereo‐seq technologies, revealing the spatiotemporal changes in gene expression profiles during human ovarian aging. Compared to the previous studies utilizing 10× Visium (Wu et al. 2024), our study presented a comprehensive and clear visualization of the human ovary. Our analyses provided four noteworthy contributions (Figure 6K). First, we mapped the spatial locations of 12 human ovarian cell types and identified aging‐related gene expression changes. Second, we uncovered the role of *CLDN5* ^+^ blood EDCs as semiprofessional APCs, which exhibited heightened inflammatory activity, contrasting with cell identity loss observed in other cell types during aging. Third, spatial mapping further uncovered immunoglobulin‐expressing cell accumulation in the ovarian periphery, correlating with advancing age. Fourth, aged human ovaries exhibited a decrease in overall cellular interactions, accompanied by an increase in *DLK1:NOTCH3* expression, which was linked to inhibited proliferation. Altogether, these findings offer novel insights into the mechanisms of human ovarian aging and suggest potential therapeutic targets for treating human disorders related to ovarian aging.

OOs were exclusively detected in the scStereo‐seq data but were absent in both our and publicly available snRNA‐seq datasets (Jin et al. 2024). For that, two potential explanations may account for this phenomenon. First, prior research has demonstrated that PAPα, an enzyme responsible for cytoplasmic mRNA polyadenylation, plays a critical role in mouse OO maturation (Jiang et al. 2021). This suggests that polyadenylated (polyA) mRNA in OOs is predominantly localized in the cytoplasm rather than the nucleus, whereas snRNA‐seq primarily captures nuclear polyA mRNA. Second, the snRNA‐seq protocol requires mechanical disruption of frozen samples to isolate nuclei. However, OOs possess fragile nuclear membranes, rendering them highly susceptible to rupture during the dissociation process, which can lead to polyA degradation or loss.

Our analysis revealed that ROS, cellular senescence, fibrosis, and DNA repair—hallmarks of aging—were broadly upregulated across cell types in aged human ovaries. We further uncovered the accumulation of immunoglobulin‐expression cells in aged human ovarian tissues. Elevated IgG levels, a key humoral immune mediator, have been implicated in deriving cellular senescence and amplifying chronic inflammation across species, including human liver and lymph nodes, as well as murine spleen, spinal cord, and small intestine (Ma et al. 2024). Beyond these established associations, our study provided evidence linking immunoglobulin dysregulation to ovarian aging, suggesting its broader role in age‐related tissue decline.

Our integrated snRNA‐seq and scStereo‐seq analyses identified a distinct blood EDC subtype, *CLDN5* ^+^ blood EDCs, which exhibited functional and transcriptional divergence from *VWF* ^+^ blood EDCs. The unique expression of MHC‐related genes, ISGs, and IFN receptors indicated their immunoregulatory potential (Amersfoort et al. 2022). Our SSS analysis further uncovered the transcriptional sensitivity of *CLDN5* ^+^ blood EDCs to aging. While their abundance remained stable across aging, these cells displayed upregulated antigen‐presenting‐related genes and enhanced inflammatory signaling, indicative of their heightened role as semiprofessional APCs in modulating local immune responses. Collectively, these findings positioned *CLDN5* ^+^ blood EDCs as critical mediators of ovarian immune homeostasis during aging.

The Notch signaling pathway is pivotal in regulating key cellular processes, including cell migration, proliferation, differentiation, and fate determination via cell‐to‐cell interactions, and plays significant physiological roles in the ovary (Guo et al. 2021). Specifically, activated Notch receptors are involved in promoting primordial follicle formation, GC proliferation, and inhibiting steroid hormone secretion (Murta et al. 2015; Prasasya and Mayo 2017). In the Notch pathway, DLK1, a noncanonical Notch ligand belonging to the EGF‐like family (Sánchez‐Solana et al. 2011), has been implicated in our study as part of the altered *DLK1:NOTCH3* axis during ovarian aging, especially in TCs and *CLDN5* ^+^ blood EDCs, where *DLK1* exhibited a progressively higher and wider expression level with age. Although the interplay of DLK1 and Notch signaling has been primarily reported in ovarian carcinogenesis, where DLK1 promotes tumorigenesis through Notch signaling activation, the relatively stable expression level of *NOTCH3* suggests that the altered *DLK1:NOTCH3* axis may have other functions during ovarian aging. A previous study demonstrated that *Dlk1* inhibits Notch signaling for the differentiation of two alveolar cell types (Finn et al. 2019). Consequently, we hypothesize that the gradual upregulation of *DLK1* inhibits *NOTCH3* during human ovarian aging, potentially disrupting normal physiological functions, including follicle development, maturation, and GC proliferation. Given this finding and the co‐occurrence of ovarian core function‐related genes and *CLDN5* ^+^ blood EDCs in SSSs, we propose that reproductive decline in aged ovaries was predominantly driven by dysregulation of the local microenvironment surrounding follicles, with relatively minimal contribution from global ovarian alterations. Further research is warranted in this area.

In summary, we have provided a comprehensive spatiotemporal single‐cell transcriptomic atlas of human ovarian aging. Our study enhances the understanding of human ovarian aging and offers a valuable resource for exploring potential therapeutic interventions.

## 4 Methods

### 4.1 Ethics

The sample collection was approved by the local ethics committee of International Peace Maternity and Child Health Hospital (Approval No. B2022269P). All participants provided informed consent and independent clinicians informed the patients about the study.

Donors in this study had a history of breast cancer treatment. Prior to ovarian reserve assessment, our hospital conducted genetic screening for *BRCA1* and *BRCA2* mutations, none of which were detected in the enrolled participants. Additionally, pathological examination of tissue sections confirmed normal histology.

### 4.2 scStereo‐Seq Sample Preparation and Sequencing

Freshly collected ovary tissue was divided into suitable blocks, and any residual liquid on the surface was promptly removed. Following this, the tissue blocks were embedded in OCT and then rapidly frozen on dry ice. The embedded samples were stored at −80°C until cryosectioning. Spatial transcriptome sequencing was performed using the STOmics Stereo‐seq transcriptome reagent set (201ST114 for 1 × 1 cm chip; 201ST004 for 0.5 × 0.5 cm chip). Tissue sections (10 μm thick) were adhered to a scStereo‐seq poly‐T carrier chip and incubated at 37°C for 4 min. Subsequently, methanol fixation, ssDNA fluorescence staining (Invitrogen, Q10212), image scanning, tissue permeabilization, reverse transcription, tissue removal, cDNA recovery, and purification were performed according to the recommended kit procedure. DNA library construction was performed using the scStereo‐seq library kit (101KL114), and qualified DNA libraries were subjected to high‐throughput sequencing on a DNBSEQ‐T7 sequencer.

### 4.3 snRNA‐Seq Library Preparation and Sequencing

The preparation of nucleus suspensions for experiments was conducted under constant ice‐cold conditions. The remaining tissue in OCT was extracted and cut in 1 mL of nucleus cell lysate (NST), which consisted of 0.1% NP40, 10 mM Tris–HCl, 146 mM NaCl, 1 mM CaCl_2_, 21 mM MgCl_2_ and 1 U/μL RNase inhibitor. The mixture was incubated on ice for 7 min to allow for cell lysis, which was confirmed by Trypan blue microscopy. After complete nucleus lysis, 1 mL of ST Wash buffer (10 mM Tris‐HCl, 146 mM NaCl, 1 mM CaCl_2_, 21 mM MgCl_2_) was added, followed by the addition of 0.01% BSA (NEB B9000S) and 40 U/mL RNase inhibitor to resuspend the nuclei. The filtrate was transferred to a 15 mL centrifuge tube through a 40 μm cell sieve (BD), rinsed with an appropriate amount of ST Wash buffer, and combined with the nuclear filtrate. The mixture was centrifuged at 500 g for 5 min at 4°C, and the nuclei were resuspended in 5 mL PBS + 1% BSA. After washing and centrifugation, the nuclei were resuspended in 100 μL PBS + 1% BSA. Finally, the nuclei were stained with Tissue Blue and counted through microscopic examination.

For snRNA‐seq, 10× Genomics Chromium Next GEM Single Cell 3′ Kit v3.1 (PN‐1000268) was used according to the User Guide (CG000315). The nucleus suspension was adjusted to an appropriate concentration (700–1200 cells/μL) and immediately loaded onto a chip to run on the 10× Chromium Controller for GEMs formation. Reverse transcription, cDNA amplification, and DNA library construction were performed according to the protocol. The concentration and fragment size of the libraries were determined separately using Invitrogen Qubit 4.0 and Agilent 4150 TapeStation, respectively. High‐throughput sequencing was performed with the PE‐150 mode.

### 4.4 Immunofluorescence Staining for Cryosections

Cryosections (8‐μm thickness) were dried at room temperature for 30 min, fixed with 4% paraformaldehyde (PFA) for 10 min, and washed three times with 1× PBS. Sections were permeabilized with 0.1% Triton X‐100 (Sigma, T8787) for 10 min at room temperature, then blocked in 1× PBS containing 5% bovine serum albumin (BSA) for 1 h at room temperature. Overnight incubation with primary antibodies was performed at 4°C in a humidified chamber using the following antibodies: STAR (1:100; Santa Cruz, sc‐166821), DLK1 (1:100; Proteintech, 10636‐1‐AP), NOTCH3 (1:100; Absin, abs116318), NOTCH2 (1:100; Proteintech, KHC1062), CLDN5 (1:100; Thermofisher, 4C3C2), IgG (1:100; Proteintech, CL488‐10284), and PDGFRA (1:50; Santa Cruz, sc‐21789). After washing with 1× PBSTx three times for 15 min each, sections were incubated with Alexa Fluor‐conjugated secondary antibodies (Invitrogen, Carlsbad, CA, USA). Following three additional washes in 1× PBSTx, sections were stained with DAPI (Beyotime, Shanghai, China) for 20 min at room temperature. Images were captured using a fluorescence microscope (Leica, Germany).

### 4.5 Immunofluorescence Staining and Quantification of Human Follicle Cells

The suspension enriched with theca cells collected from human follicular fluid was placed onto a glass slide and allowed to settle for 2–3 h. The upper suspension was then carefully aspirated and the slide was rinsed once with PBS. Cells were immediately fixed with 4% PFA. The subsequent procedures were consistent with the staining protocol for cells on slides. Details on the antibodies and their working concentrations are as follows: STAR (1:100; Santa Cruz, sc‐166821), and DLK1 (1:100; Proteintech, 10636‐1‐AP).

The method for quantifying immunofluorescence means optical density (Mean Density) involves the following steps: For each section, at least three fields of view at 200× magnification are randomly selected to ensure comprehensive sampling. During image capture, the tissue should fill the entire field of view to maintain consistent background illumination. Images are processed with software *Image‐Pro Plus 6.0*, which converts green and red fluorescence images to grayscale. A uniform shade of black is established as the standard to evaluate positivity across all images. The integrated optical density (IOD) and the pixel area (AREA) of the tissue in each image are then analyzed to calculate the Mean Density.

### 4.6 Cell Culture of the Human GC Cell Line (SVOG)

The cells were seeded (0.5 ~ 2 × 10^5^ cells/mL in 24‐well plates) and cultured in DMEM/F12 medium (Thermo Fisher, 11320) containing 10% FBS (Hyclone), 1% penicillin and streptomycin (Thermo Fisher, 15140‐122). Culture medium was changed every other day in all experiments. Cultures were maintained at 37°C in a humidified atmosphere containing 5% CO_2_ and 95% air.

### 4.7 Overexpression of *DLK1* in SVOG Cell Line

For *DLK1* overexpression, a plasmid construct encoding human *DLK1* was generated. Cells were seeded into 24‐well plates 1 day before transfection to reach 70%–90% confluence. Transfection was carried out using Lipofectamine 3000 (Invitrogen) following the manufacturer's protocol. Briefly, 500 ng plasmid DNA was diluted in 50 μL Opti‐MEM medium (Thermo Fisher, 31985070) and mixed with 1 μL P3000 reagent. In parallel, 1.5 μL Lipofectamine 3000 reagent was diluted in 25 μL Opti‐MEM medium. The diluted DNA/P3000 mixture was then combined with the diluted Lipofectamine 3000 reagent (1:1), mixed gently, and incubated for 10–15 min at room temperature to allow complex formation. Subsequently, 50 μL of the DNA–lipid complex was added dropwise to each well containing cells in fresh culture medium. After 48 h of incubation, cells were collected and lysed in TRIzol reagent for RNA extraction. The sequence of the *DLK1* overexpression plasmid was provided as follows, DLK1‐F (atgaccgcgaccgaa) and DLK1‐R (ttagatctcctcgtc).

### 4.8 Collection of Human Primary GCs

Human primary GCs were collected from the cumulus‐oocyte complexes (COCs) remaining after oocyte retrieval. The cumulus cells were dissociated by 0.05% Trypsin‐EDTA (Thermo Fisher, 25200056), followed by centrifugation to pellet the cells. The resulting GCs were immediately lysed in TRIzol reagent for RNA extraction.

### 4.9 RNA Extraction and Quantitative Real‐Time PCR Analysis

Total RNAs from isolated human primary GCs were extracted with Trizol following the manufacturer's protocol. The cDNA was generated from equal amounts of RNA using the SuperScriptIII kit. The expression level of selected genes was validated and qPCR was performed on a StepOne Plus (Applied Biosystems) with Power SYBR Green PCR Master Mix. RNA level was normalized to *GAPDH*. The cycling condition was 10 10 min at 95°C for initial denaturing, 40 cycles of 15 s at 95°C for denaturing, 1 min at 60°C for annealing and extension, followed by a melt curve test. The primers used in this were shown as follows.

**Table:** 

Hu‐NOTCH3‐F	TGGCGACCTCACTTACGACT
Hu‐*NOTCH3*‐R	CACTGGCAGTTATAGGTGTTGAC
Hu‐*DLK1*‐F	TCCTCAACAAGTGCGAGACC
Hu‐*DLK1*‐R	AGATGATGTTGACGGCCAGG
Hu‐*p*16 (*CDKN2A*)‐F2	CATGGAGCCTTCGGCTGAC
Hu‐*p16* (*CDKN2A*)‐R2	TCATCATGACCTGGATCGGC
Hu‐*p21* (*CDKN1A*)‐F1	GCGACTGTGATGCGCTAATG
Hu‐*p21* (*CDKN1A*)‐R1	GAAGGTAGAGCTTGGGCAGG

### 4.10 snRNA‐Seq Data Processing

The raw snRNA‐seq data were processed using 10× Genomics software *CellRanger* (version: 7.0.1), in which process the raw sequencing data were mapped to the human reference genome (GRCh38).

After obtaining processed snRNA‐seq data, we applied quality control measures to retain the high‐quality single nucleus/cell for downstream analysis. Using the R package *Seurat* (version: 5.0.2) (Stuart et al. 2019), we filtered out empty droplets with fewer than 200 detected genes expressed in at least 3 cells. We further saved single cells based on the number of detected genes (200–10,000), the number of detected UMIs (1000–30,000), and the percentage of mitochondrial UMIs (< 20%). To remove potential doublets, we utilized the Python package *scrublet* (Wolock et al. 2019), identifying and filtering out cells with a *doubletScore* greater than the 90% quantile. Finally, we retained high‐quality single cells with more than 500 detected genes and more than 1000 detected UMIs for subsequent analysis.

The gene expression level of snRNA‐seq data was quantified as UMI counts per million mapped reads (CPM), calculated by dividing the UMI count of a given gene in a single cell by the total UMI count of that cell and multiplying by 100,000. The gene expression level of snRNA‐seq data was then transformed into log_2_(CPM + 1).

For human ovaries, we obtained the processed 10× snRNA‐seq and scRNA‐seq data from Gene Expression Omnibus (GEO) under the accession code GSE202601 (Jin et al. 2024) and GSE255690 (Wu et al. 2024), respectively. We retained high‐quality single cells using the same standard as described above. For mouse and NHP ovaries, we obtained the processed scRNA‐seq data (with cellular annotation information) from GEO under the accession code GSE232309 (Isola et al. 2024) and GSE130664 (Wang et al. 2020), respectively.

### 4.11 Cell Type Identifying by snRNA‐Seq Data

To identify cell types based on snRNA‐seq data from 12 human ovaries, we loaded the data quantified with raw UMI counts into *Seurat*. For each sample, the UMI counts were separately normalized using the function *NormalizeData* in *Seurat* with default parameters, loaded to identify highly variable genes (HVGs) using the function *FindVariableFeatures* with default parameters, and scaled using the function *ScaleData* with all detected genes. We then performed principal component analysis using the function *RunPCA*. To minimize the bias of individual heterogeneity, we applied the Harmony algorithm using the function *IntegrateLayers* (Korsunsky et al. 2019), which converged after three iterations. After that, samples were merged using the function *JoinLayers*. According to selected Harmony dimensions, UMAP nonlinear dimensionality reduction analysis and unsupervised clustering analysis were performed using the function *RunUMAP* and *FindClusters* with default parameters, respectively. Finally, based on the expression profiles of lineage well‐known marker genes, 12 cell types were defined.

To validate ovarian cell annotation, we collected gene signatures of publicly annotated cell types (Fan et al. 2019), and calculated the gene signature module score using function *AddModuleScore* in *Seurat*.

### 4.12 Cell‐Type‐Specific Marker Genes Identifying by snRNA‐Seq Data

We identified cell‐type‐specific marker genes that were also DEGs across diverse cell types. The snRNA‐seq data quantified with raw UMI counts was applied, and these DEGs were detected using a Wilcoxon rank sum test through function *FindAllMarkers* in *Seurat*. Genes were considered DEGs if they met the following criteria: a log_2_‐transformed fold change exceeding 0.25, a false discovery rate (FDR) below 0.05, and an expression percentage in the corresponding cell type surpassing 25%. In visualizing the expression patterns of these cell‐type‐specific marker genes, we determined the mean expression level of each gene within a cell type, and then normalized it to a scale ranging from 0 to 1 across all cell types. To gain functional insights into cell‐type‐specific marker genes, GO analysis was performed using the web tool *Metascape* with default parameters (Zhou et al. 2019).

We identified cell‐type‐specific marker genes of EDC subtypes using the same method and parameters as the above description.

### 4.13 Establishment of the Cell Scoring Model Based on the Machine‐Learning Method

To assess the presence of oocytes within the snRNA‐seq dataset, we developed a cell scoring model based on the well‐defined scRNA‐seq data from NHP ovaries (Wang et al. 2020), following a similar methodology to a previous report (La Manno et al. 2016). Firstly, we calculated the expression pattern distribution of well‐annotated NHP ovarian cells (Wang et al. 2020), and used this as a reference to map our human ovarian single cells.

Briefly, we trained the cell scoring model using publicly available scRNA‐seq data from NHP ovaries (Wang et al. 2020). Gene expression levels of this public data were quantified as CPM. To minimize the influence of housekeeping genes, we selected the top 3000 HVGs based on coefficient of variation–mean values. A logistic regression model with L2‐norm regularization and a multinomial learning approach was trained on log_2_‐transformed, max‐normalized data using function *LogisticRegression* in Python package *scikit‐learn* (Pedregosa et al. 2011). This trained model served as a reference to predict the probabilities of cell type assignments for our single‐cell data which were also quantified as CPM. Predicted probabilities were computed using function *softmax*.

To further classify oocyte subtypes, we established a separate cell scoring model using scRNA‐seq data exclusively from NHP oocytes (Wang et al. 2020). The model parameters were the same as the above description.

### 4.14 Cross‐Species Comparison Between Human and Mouse Ovaries

Using a multi‐class classification approach, we established cross‐species cell type correspondences between human and mouse ovarian cells, as previously described (Peng et al. 2019; Shekhar et al. 2016). Briefly, cross‐species analysis was performed based on normalized snRNA‐seq count data using the R package *Seurat*. The dataset included 7842 single cells from young mice and 5489 from old mice. We separately analyzed transcriptomic correlations within young and old groups. For each species, we identified 3000 HVGs using the function *FindVariableFeatures* in *Seurat*. To ensure accurate comparisons, we mapped homologous genes between human and mouse, excluding genes with one‐to‐multiple correspondences (NCBI Resource Coordinators 2016). A multi‐class classification model was trained using the Xgboost algorithm, implemented via the R package *xgboost* (version: 1.7.8.1). For each comparison (young human versus young mouse, or old human versus old mouse), based on the common HVGs (999 and 962 common HVGs for the young and old groups, respectively), we used 80% of human cells from each cell type (up to 1000 cells per cell type) to train the model, with the remaining cells reserved for testing. The random seed was set to “20010117” for reproducibility. The Adjusted Rand Index values for the young and old human cells were 0.93 and 0.97, respectively, indicating robust prediction accuracy with minimal overfitting. Using the trained models, we assigned each mouse cell to a human ovarian cell type.

### 4.15 Calculation of Cell Identity by snRNA‐Seq Data

To quantify cell identity, we first identified cell‐type‐specific marker genes (DEGs) across diverse cell types in the young group. The snRNA‐seq data, quantified using raw UMI counts, were analyzed using the function *FindAllMarkers* in *Seurat*, employing a Wilcoxon rank sum test. DEGs were defined based on the following criteria: a log_2_‐transformed fold change exceeding 1, an FDR below 0.05, and expression percentage in the corresponding cell type surpassing 25%. We defined these DEGs as the “cell identity gene”.

Next, we selected the top 50 cell identity genes for each cell type based on the fold change, and calculated the gene module score (we termed this as the “cell identity score”) for each single cell using the function *AddModuleScore* in *Seurat*. For each cell type within a given age group, we calculated the average gene module score and determined its increase or decrease level relative to the young group.

### 4.16 Aging‐Related DEGs Identifying by snRNA‐Seq Data

The log_2_‐transformmed CPM snRNA‐seq data were utilized to identify aging‐related DEGs using the function *FindAllMarkers* in *Seurat*, employing a Wilcoxon rank sum test. Aging‐related DEGs were defined based on the following criteria: a log_2_‐transformed fold change exceeding 0.25, an FDR below 0.05, and an expression percentage in the corresponding age group surpassing 25%. GO analysis was performed using the web tool *Metascape* with default parameters.

### 4.17 scStereo‐Seq Data Processing, and Cell Types and Cell‐Type‐Specific Marker Genes Identifying

The scStereo‐seq analysis workflow (SAW) (version: 7.0.0) provided by The Beijing Genomics Institute (BGI) was used to process scStereo‐seq spatial RNA‐seq output and brightfield microscope images in order to detect tissue, map reads to the human reference genome (GRCh38) using software *STAR* (Dobin et al. 2013), generate feature‐CID (coordinate identifier) matrices, perform clustering and gene expression analysis, and place spots in spatial context on the slide image.

For the downstream analysis, we transferred the file format of “*tissue.gtf*” to “*rds*” using function *io.stereo_to_anndata* and the public script *h5ad2rds.R* in the Python package *Stereopy* (version: 1.5.1). For balancing the single‐cell resolution and captured genes, we applied bin 50 (50 × 50 bins) as the spot to analyze the ovary tissue slice (Chen et al. 2022).

The transferred *Seurat* objects were loaded into the R environment; only cells with more than 200 genes were retained for the downstream analysis. For each sample, the scStereo‐seq data was separately processed using the function *SCTransform* with a parameter “method = glmGamPoi” in *Seurat* (version: 4.3.0). We then selected 3000 features for further integration using the function *SelectIntegrationFeatures*, detected anchors using the functions *PrepSCTIntegration* and *FindIntegrationAnchors*, and lastly integrated scStereo‐seq data using the function *IntegrateData*. The same processing methods were also applied to the snRNA‐seq data. To identify the cell type in scStereo‐seq data, we then transferred the cell annotation obtained from snRNA‐seq data into scStereo‐seq data. Of note, although we reprocessed the snRNA‐seq data here using *Seurat* (version: 4.3.0), we still utilized the cell annotation information obtained based on *Seurat* (version: 5.0.2) for transferring. We identified anchors using the function *FindTransferAnchors*, transferred data using the function *TransferData*, and finally annotated cell types using the function *AddMetaData*.

The scStereo‐seq data quantified with raw counts was applied to identify cell‐type‐specific marker genes (DEGs across cell types), and these DEGs were detected using a Wilcoxon rank sum test through the function *FindAllMarkers* in *Seurat*. DEGs were defined based on the following: a log_2_‐transformed fold change exceeding 0.25, an FDR below 0.05, and an expression percentage in the corresponding cell type surpassing 10%. GO analysis was performed using the web tool *Metascape* with default parameters.

### 4.18 Gene Module Score Analysis of Aging Hallmarks by scStereo‐Seq Data

To quantify the expression pattern of classic aging hallmarks, we collected gene sets from a previous study (Bao et al. 2023). For each single cell, we calculated the gene module score using the function *AddModuleScore* in *Seurat*.

### 4.19 SSS Analysis

To comprehensively characterize the ovarian microenvironment within senescence hotspots, we identified SSSs as a previously established methodology (Ma et al. 2024). Briefly, we integrated five well‐defined senescence gene sets from prior studies (Avelar et al. 2020; Fridman and Tainsky 2008; Aging Atlas Consortium 2021; Saul et al. 2022). Using log_2_‐transformed CPM snRNA‐seq data (without cell type separation), we identified our aging‐related DEGs between the older‐aged and young groups, applying the same methods and parameters as described above. We then intersected the senescence gene sets with our aging‐related upregulated DEGs to identify aging‐sensitive genes. For scStereo‐seq data, we calculated the gene module score of our defined aging‐sensitive genes using the function *AddModuleScore* in *Seurat*. For the ovary at age 42, we defined the top‐ranked 5% of spots with the highest gene module scores of aging‐sensitive genes as SSSs.

The log_2_‐transformmed CPM scStereo‐seq data were utilized to identify DEGs between SSSs and other spots using the function *FindAllMarkers* in *Seurat*, employing a Wilcoxon rank sum test. DEGs were defined based on the following criteria: a log_2_‐transformed fold change exceeding 0.25, an FDR below 0.05, and an expression percentage in the corresponding age group surpassing 10%. GO analysis was performed using the web tool *Metascape* with default parameters.

### 4.20 Co‐Occurrence Analysis by scStereo‐Seq Data

We conducted spatial co‐occurrence analysis of diverse cell types using function *co_occurrence* in *Stereopy* with the parameter “method=stereopy, dist_thres=180, steps=6”. We displayed co‐occurrence probability under the distance threshold of 60 using function *co_occurrence_heatmap* in *Stereopy*.

### 4.21 Spatial Hotspot Identification by scStereo‐Seq Data

The raw scStereo‐seq counts were utilized to identify spatial hotspots. The count data were normalized, transformed, and scaled using functions *normalize_total*, *log1p*, and *scale* in *Stereopy*, and then HVGs were selected using function *highly_variable_genes* in *Stereopy* with the parameters “min_mean=0.0125, max_mean=3, min_disp=0.5, n_top_genes=2000”. We identified spatial hotspots using function *spatial_hotspot* in *Stereopy* with the parameters “use_highly_genes=True, use_raw=True, model=normal, n_neighbors=30, fdr_threshold=0.05, min_gene_threshold=10”. Functions *hotspot_local_correlations* and *hotspot_modules* in *Stereopy* were applied for the visualization of spatial hotspots.

### 4.22 Cell–Cell Interaction Analysis by scStereo‐Seq Data

We applied scStereo‐seq data to perform the cell–cell interaction analysis using *Stereopy*, and this analysis was conducted independently for each age. In this analysis, we applied the analysis type as “*statistical*” type and the “*cellphonedb*” database using the function *tl.cell_cell_communication* with other default parameters.

### 4.23 Statistical Analysis

For bioinformatics data, two‐tailed Student's *t*‐test, one‐tailed Wilcoxon‐ranked sum test, and R software were used for statistical analysis. A value of *p* < 0.05 or FDR < 0.05 was considered to indicate statistical significance.

## Author Contributions

Wen Li, Yuxuan Zheng, Ningxia Sun, and Meiling Zhang initiated and coordinated the project. Yuxuan Zheng performed bioinformatics analysis. Meiling Zhang, Fanghao Guo, and Qing Zhang performed immunofluorescence staining and collected human ovarian samples with the help of Di Sun, Yongjian Ma, Yanquan Li, Mengxi Guo, Haixia Ding, Ying Guo, Baicai Yang, and Songmao Li. Qianhui Hu performed in vitro cell experiment. Yuxuan Zheng and Fanghao Guo wrote and polished the manuscript.

## Conflicts of Interest

The authors declare no conflicts of interest.

## Supporting information


**Figure S1:** Quality control information of snRNA‐seq data. (A) Representative H&E staining of human ovarian in different age‐stage for scStereo‐seq. Scale bar, 1000 μm. (B) Violin plots showing the number of detected genes (top) and UMIs (middle), and the percentage of mitochondrial transcripts (bottom). (C) UMAP plot showing the distribution of cells colored by donor information. The donor information is named as “age group + donor ID + study source”, in which “O” and “J” indicate “our study” and “Jin's study”, respectively. The snRNA‐seq of Jin's study is collected from the previous study (Jin et al. 2024). (D) Heatmap showing the row‐scaled gene module score of previously defined ovarian gene signatures collected from the previous study (Fan et al. 2019). (E) UMAP plot showing the cellular annotation provided by the previous study (Jin et al. 2024). (F) Dot plot showing the expression profiles of cell‐type‐specific marker genes among snRNA‐seq cell types. Dot size indicates the percentage of expressed cells and dot color indicates the scaled expression level. For each gene, the expression level is scaled among cell types. (G) Scatter plots showing the distribution of single cells in the established machine‐learning model. Single cells are collected from the public scRNA‐seq (Wang et al. 2020) (left) and this study (right). (H) Dot plots showing the percentage of mouse young (left) and old (right) ovarian cells corresponding to human young (left) and old (right) ovarian cells.
**Figure S2:** Quality control information of scStereo‐seq data. (A) Spatial visualizations showing the quality control information of scStereo‐seq data. (B) Bar plots showing GO terms of cell‐type‐specific marker genes revealed by scStereo‐seq data, corresponding to Figure 1F. (C and D) Immunofluorescence staining of STAR in the human ovary. Scale bar, 50 μm. (E) Bar plot showing the relative number of STAR^+^ cells within the human ovary at ages 32, 34, and 42, compared to age 12. Two‐tailed Student's *t*‐test is performed, in which ***p < 0.001. Data are shown as mean ± SEM. (F) Spatial visualizations showing the expression level of well‐known marker genes in TCs (*STAR*) and SCs (*PDGFRA*). The scale of color bars is varied among ages and genes. (G) Dot plot showing the expression profiles of the SC specific marker (*PDGFRA*) among scStereo‐seq cell types. Dot size indicates the percentage of expressed cells and dot color indicates the scaled expression level. For this gene, the expression level is scaled among cell types. (H) Heatmap showing the row‐scaled gene module score of ovarian gene signatures (marker genes) in distinct cell types identified by scStereo‐seq data. These ovarian gene signatures were identified by snRNA‐seq data (Figure S1F; Table S2). (I) Immunofluorescence staining of STAR and PDGFRA in the human ovary at age 42. The white and yellow arrow indicate SCs (STAR^+^PDGFRA^+^) in the form of clumps and TCs (STAR^+^PDGFRA^−^) surrounding follicles, respectively. Scale bar, 50 μm.
**Figure S3:** Gene expression profiles in scStereo‐seq data. (A) Spatial visualizations showing the expression level of well‐known marker genes in distinct cell types. The scale of color bars is varied among ages and genes. (B) Bar plot showing the cell number of ovarian cell types corresponding to scStereo‐seq data. The percentage of diverse cell types are indicated in brackets. (C) Scatter plots showing the distribution of oocytes in the established machine‐learning model. Oocytes are collected from the public scRNA‐seq (Wang et al. 2020) (top) and this study (bottom). (D) Separated spatial visualizations showing the spatial distribution of cell types at ages 12, 32, 34, and 42.
**Figure S4:** Dynamic expression profiling of aging hallmarks during ovarian aging. (A) Left, spatial visualizations showing the expression level of *CDKN2A*. Representative regions are zoomed in. Right, bar plot showing the percentage of *CDKN2A* ^+^ spots among all ovarian spots at diverse ages. (B) Boxplots showing the gene module score of representative aging hallmarks. Two‐tailed Student's *t*‐test *p* values are indicated, in which ***p < 0.001. (C) Boxplot showing the gene module score of antioxidant genes. Two‐tailed Student's *t*‐test *p* values are indicated, in which ns, not significant, **p < 0.01, and ***p < 0.001. (D) Dot plots showing GO terms corresponding to the aging‐related upregulated DEGs between middle‐aged and young groups (left), older‐ and middle‐aged groups (middle), or older‐aged and young groups (right). Sizes and colors indicate GeneRatio and significant statistic of GO terms, respectively. (E) Dot plots showing the log_2_‐transformmed fold change of overlapped genes between aging‐related DEGs and POF/POI‐related genes. These aging‐related upregulated (left) and downregulated (right) DEGs are identified between older‐aged and young groups. (F) Bar plots showing the expression levels of representative genes within *CYP19A1* ^+^ GCs based on snRNA‐seq data (left) or oocytes based on scStereo‐seq data (right). Two‐tailed Student's *t*‐test *p* values are indicated, in which ns, not significant, *p < 0.05, **p < 0.01, and ***p < 0.001. Data are shown as mean ± SEM. (G) Dot plot showing GO terms corresponding to the aging‐related upregulated DEGs between young and prepuberal groups. Sizes and colors indicate GeneRatio and significant statistic of GO terms, respectively.
**Figure S5:** Activated inflammatory microenvironment in aged human ovaries. (A) Volcano plot showing the aging‐related DEGs between older‐aged and young groups (without cell type separation) based on snRNA‐seq data. The numbers of aging‐related upregulated and downregulated DEGs are indicated in brackets. Representative DEGs are indicated. (B) Bar plots showing GO Biological Process (top) and Wiki Pathway (bottom) terms corresponding to upregulated DEGs between SSSs and other spots (with cell type separation). Only upregulated DEGs occur in at least three cell types are considered, corresponding to Figure 4J.
**Figure S6:** Ovarian spatial hotspots at diverse ages. (A) Heatmaps showing the correlation among genes assigned into diverse spatial hotspots, according to scStereo‐seq data. (B–E) Left, spatial visualizations showing the expression level of spatial hotspots at ages 12 (B), 32 (C), 34 (D), and 42 (E). Right, dot plots showing GO terms corresponding to spatial hotspots at ages 12 (B), 32 (C), 34 (D), and 42 (E). Sizes and colors indicate GeneRatio and significant statistic of GO terms, respectively.
**Figure S7:** Dynamic cell–cell interactions among ovarian cell types during aging. (A) Cell–cell interaction networks showing interactions between any two cell types. The dot color indicates the cell type, the dot size indicates the interaction number of a given cell type, and the line thickness indicates the interaction number of a given cell type pair. (B) Spatial visualizations showing the expression level of *DLK1* and *NOTCH3*. The scale of color bars is varied among ages and genes. (C) Bar plots showing the expression level of *DLK1* and *NOTCH3* across cell types and ages. Data are shown as mean ± SEM. (D) Bar plot showing the expression level of *DLK1* within *STAR* ^+^ cells in human ovaries, according to scRNA‐seq data collected from the previous study (Wu et al. 2024). Two‐tailed Student's *t*‐test *p* values are indicated, in which ***p < 0.001. Data are shown as mean ± SEM.

**Table S1:** acel70288‐sup‐0002‐TableS1.xlsx.

**Table S2:** acel70288‐sup‐0003‐TableS2.xlsx.

**Table S3:** acel70288‐sup‐0004‐TableS3.xlsx.

**Table S4:** acel70288‐sup‐0005‐TableS4.xlsx.

**Table S5:** acel70288‐sup‐0006‐TableS5.xlsx.

## Data Availability

The raw snRNA‐seq and scStereo‐seq data reported in this study have been deposited in Genome Sequence Archive (GSA) for Human at https://ngdc.cncb.ac.cn/gsa‐human/, reference number HRA010703. The processed sequencing data that support the findings of this study are openly available in Gene Expression Omnibus (GEO) at https://www.ncbi.nlm.nih.gov/geo/, reference number GSE267315.
