# Supplementary figures and images for: Spatial Transcriptomic Characteristics of the Aging Human Ovary
Source: Aging Cell. 2025 Nov 17;25(1):e70288. doi: 10.1111/acel.70288 (PMC12740089; doi:10.1111/acel.70288)

Figure S1

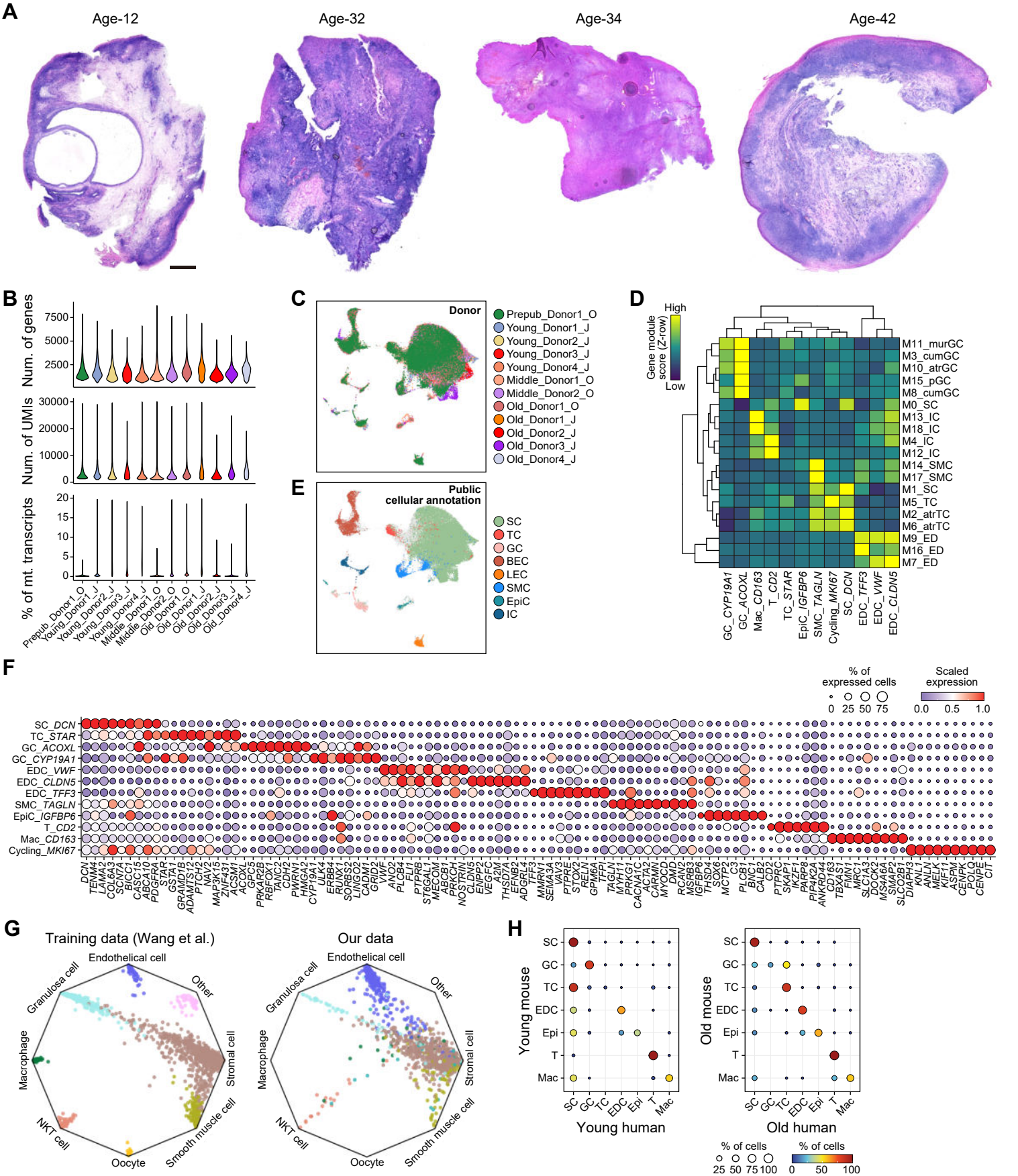

Figure S2

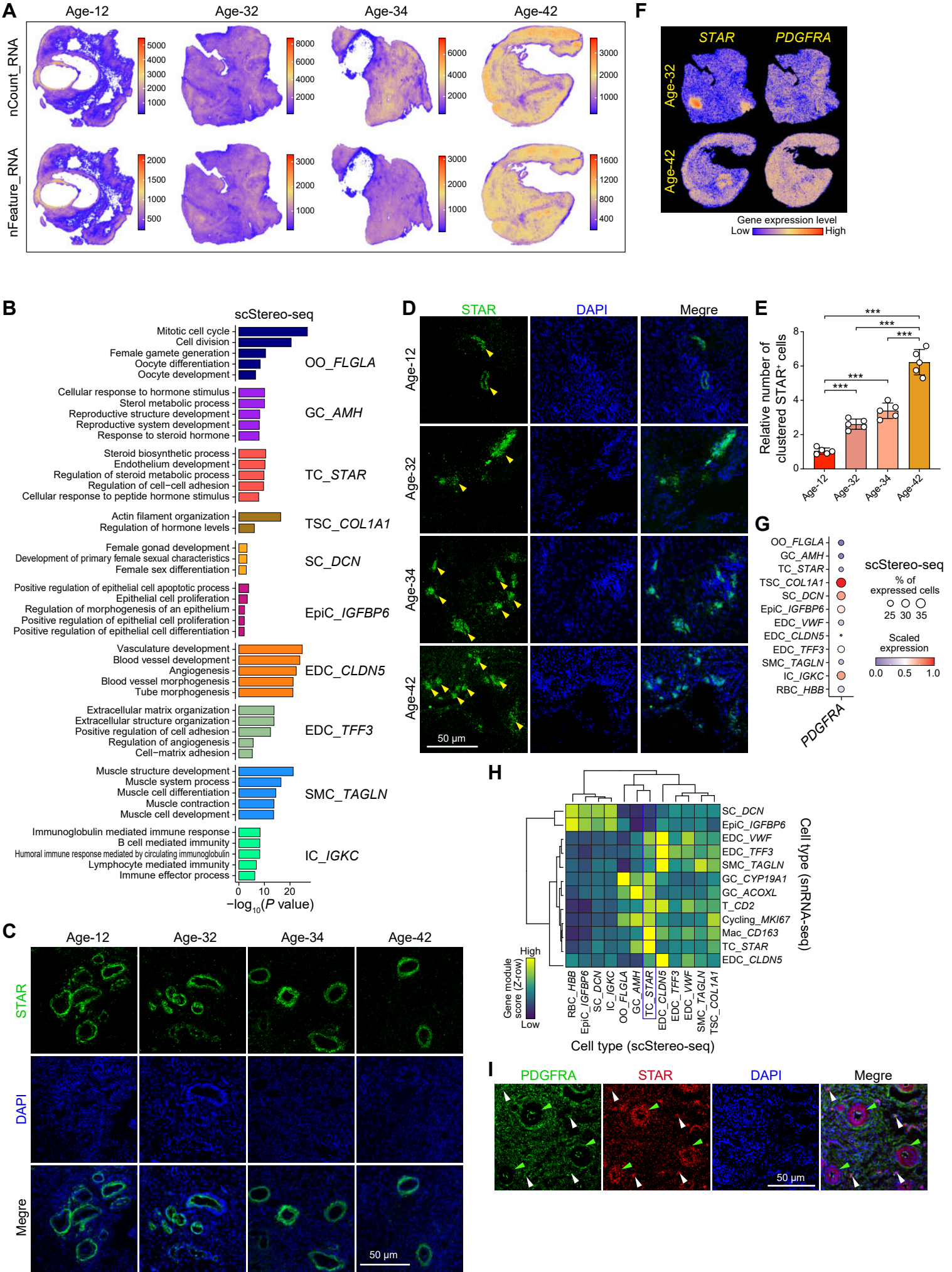

Figure S3

A

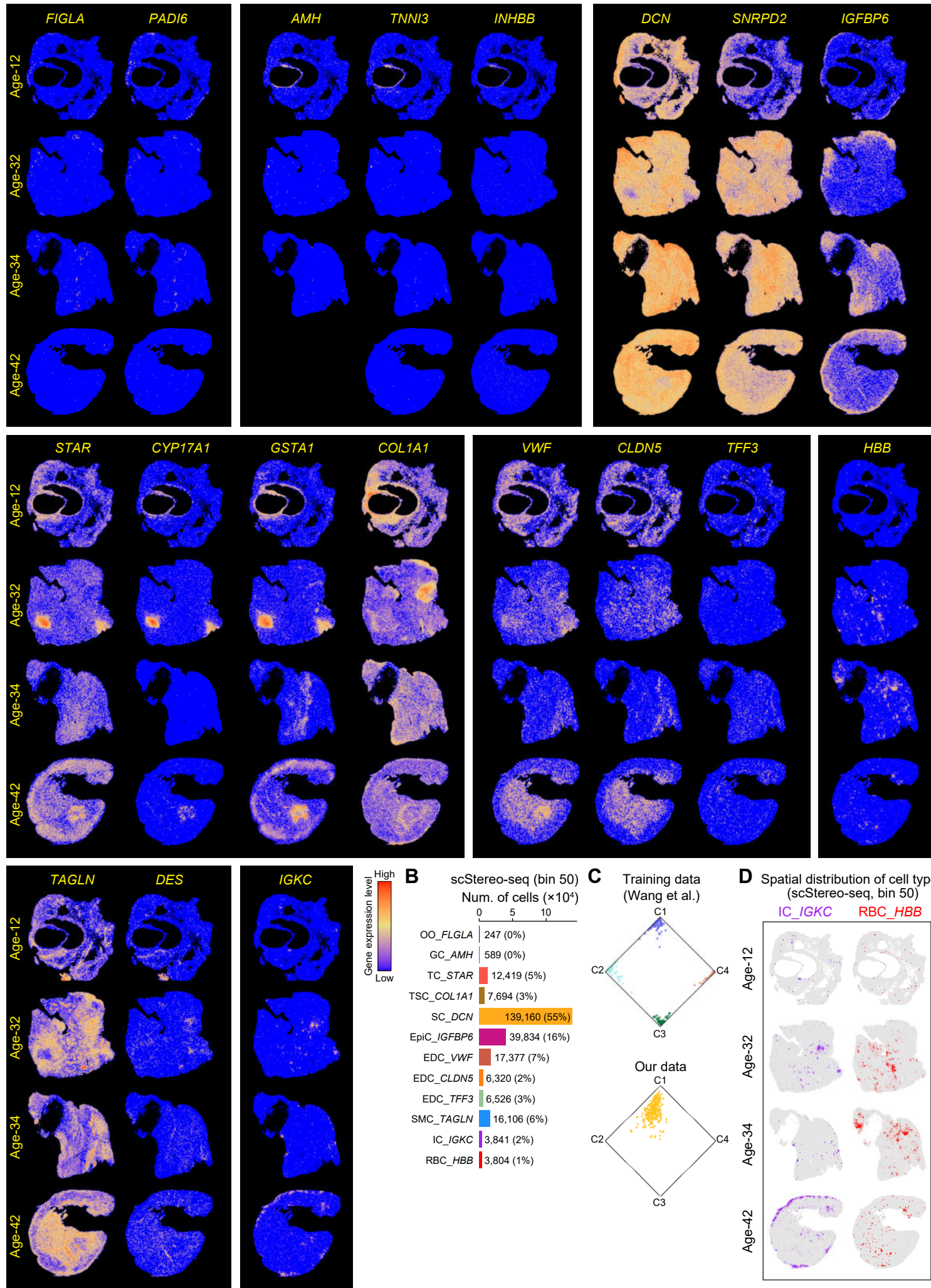

Figure S4

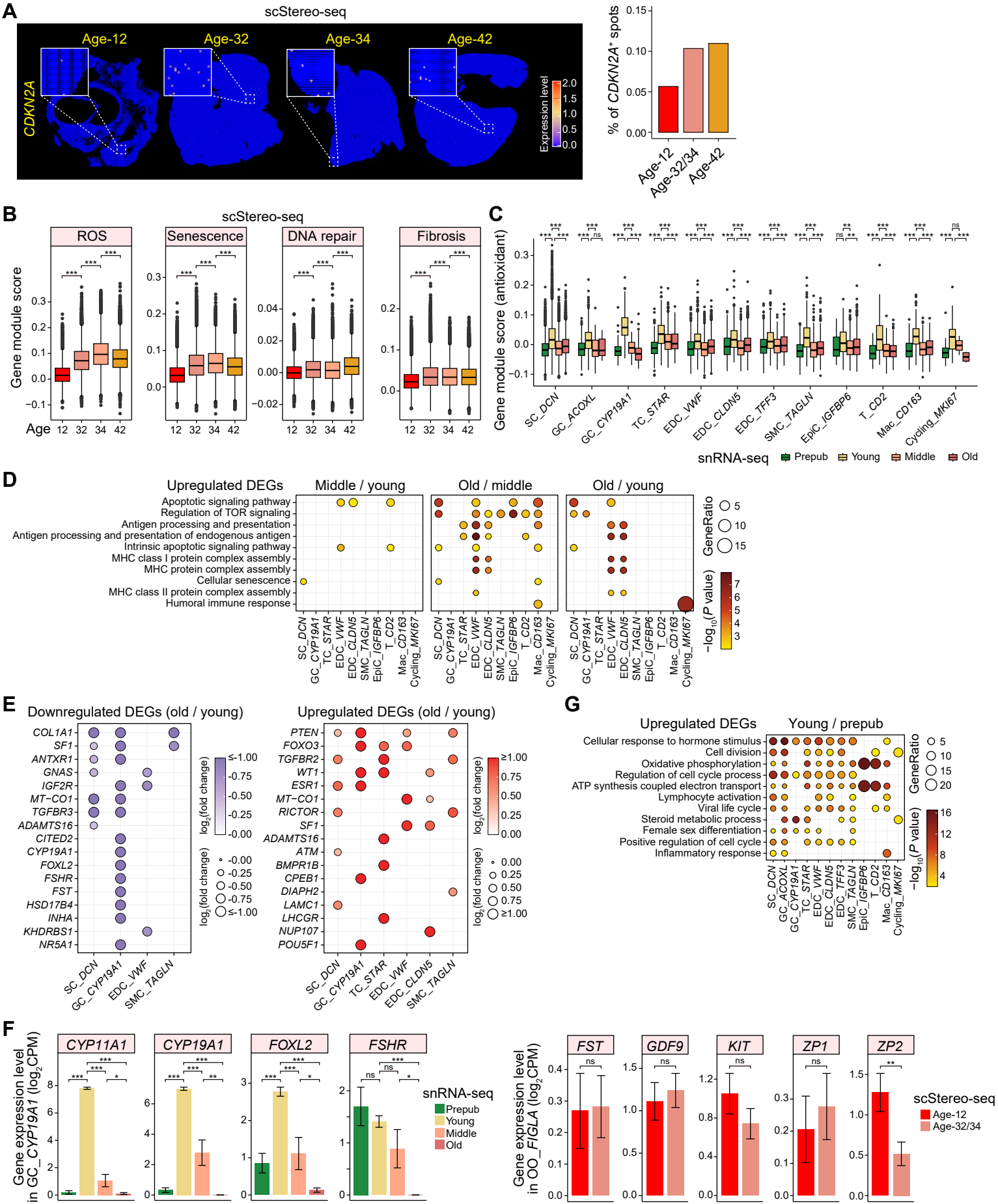

Figure S5

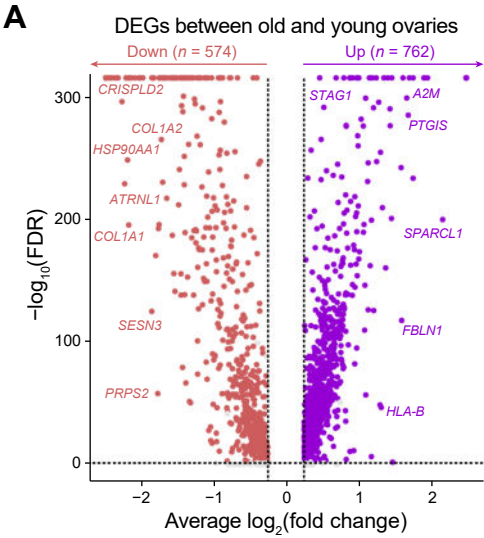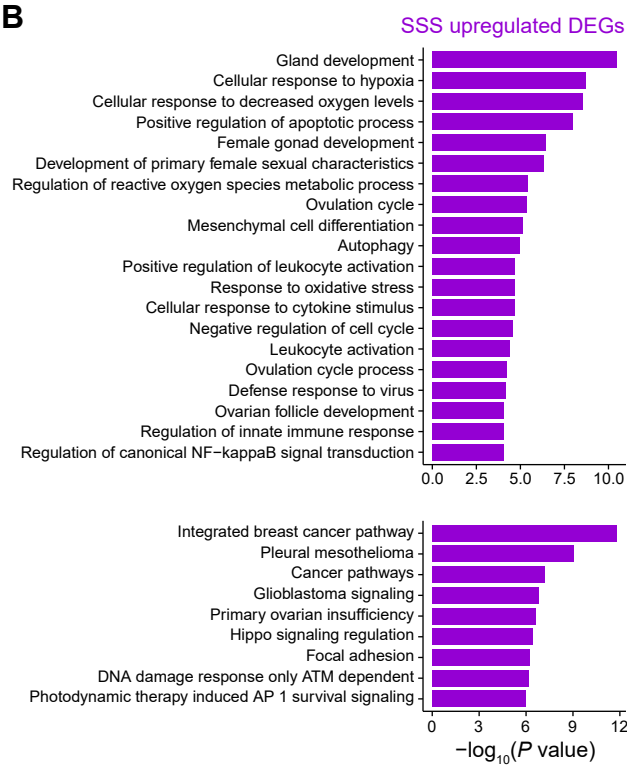

Figure S6

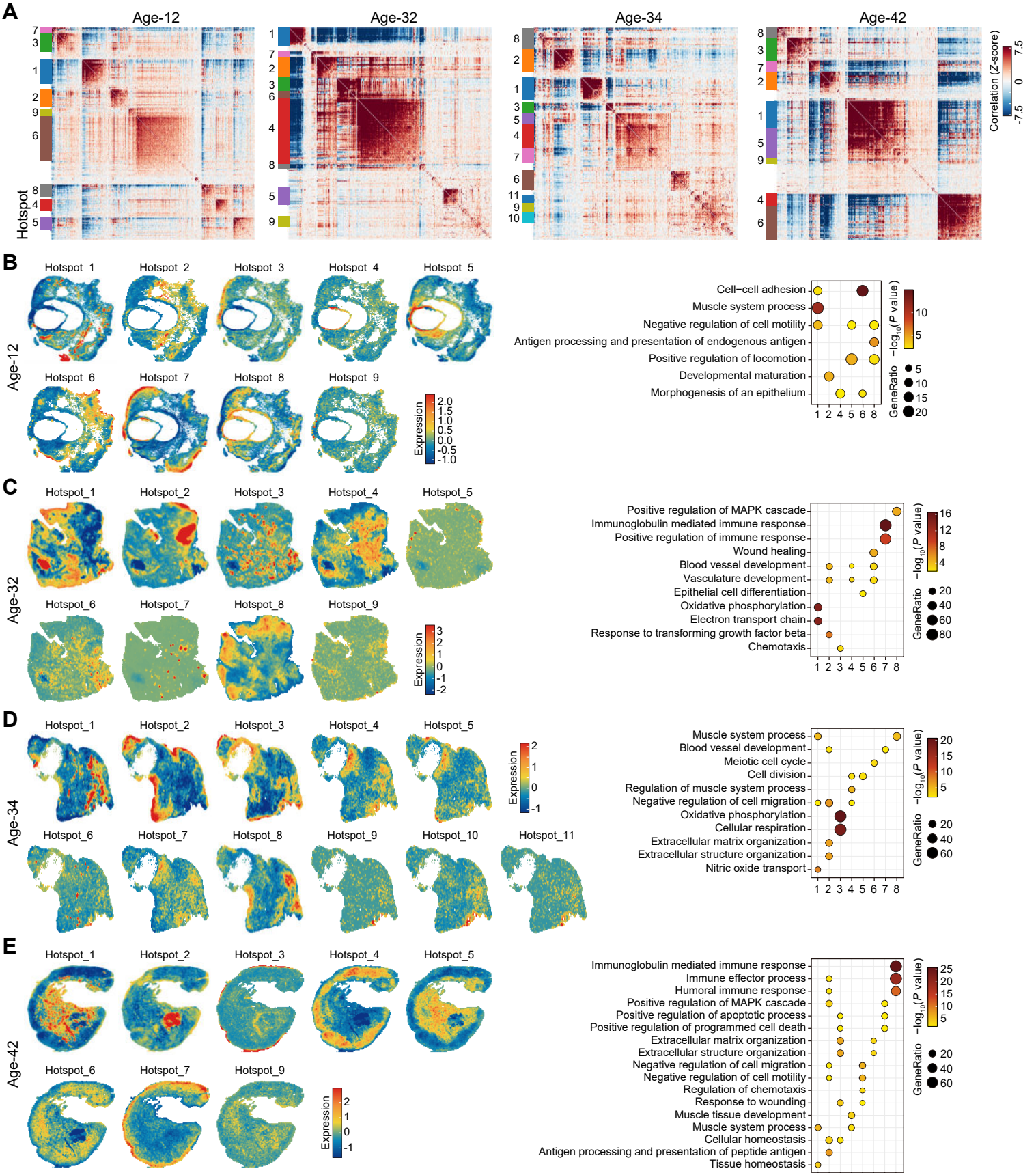

Figure S7

A

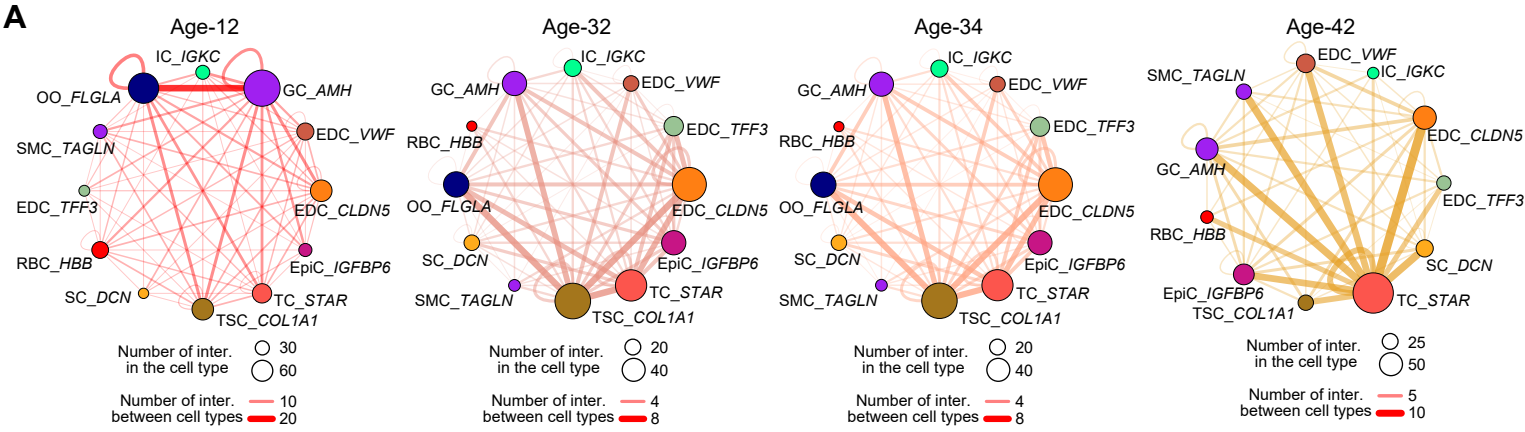

B

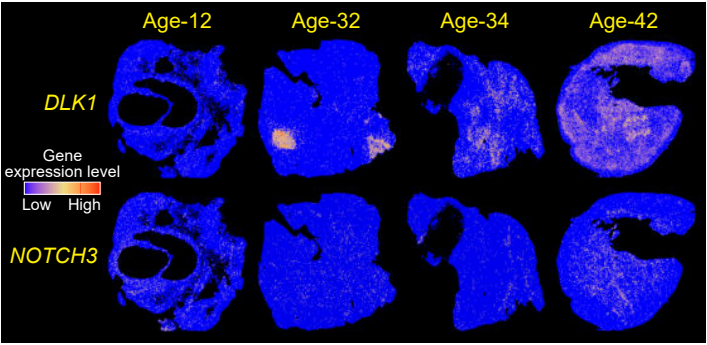

C

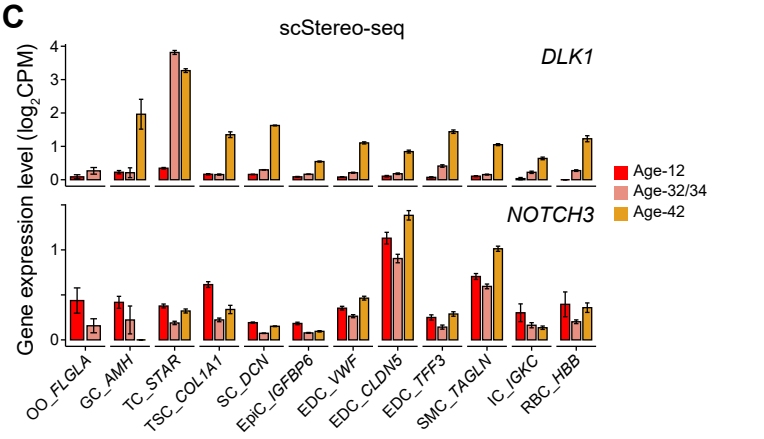

D

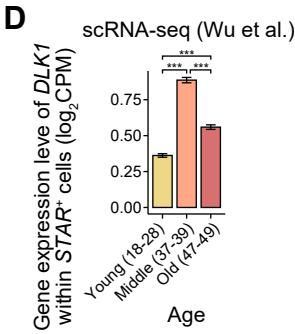

Supplement: Supplementary file 1 — Figure S1: Quality control information of snRNA‐seq data. (A) Representative H&E staining of human ovarian in different age‐stage for scStereo‐seq. Scale bar, 1000 μm. (B) Violin plots showing the number of detected genes (top) and UMIs (middle), and the percentage of mitochondrial transcripts (bottom). (C) UMAP plot showing the distribution of cells colored by donor information. The donor information is named as “age group + donor ID + study source”, in which “O” and “J” indicate “our study” and “Jin's study”, respectively. The snRNA‐seq of Jin's study is collected from the previous study (Jin et al. 2024). (D) Heatmap showing the row‐scaled gene module score of previously defined ovarian gene signatures collected from the previous study (Fan et al. 2019). (E) UMAP plot showing the cellular annotation provided by the previous study (Jin et al. 2024). (F) Dot plot showing the expression profiles of cell‐type‐specific marker genes among snRNA‐seq cell types. Dot size indicates the percentage of expressed cells and dot color indicates the scaled expression level. For each gene, the expression level is scaled among cell types. (G) Scatter plots showing the distribution of single cells in the established machine‐learning model. Single cells are collected from the public scRNA‐seq (Wang et al. 2020) (left) and this study (right). (H) Dot plots showing the percentage of mouse young (left) and old (right) ovarian cells corresponding to human young (left) and old (right) ovarian cells. Figure S2: Quality control information of scStereo‐seq data. (A) Spatial visualizations showing the quality control information of scStereo‐seq data. (B) Bar plots showing GO terms of cell‐type‐specific marker genes revealed by scStereo‐seq data, corresponding to Figure 1F. (C and D) Immunofluorescence staining of STAR in the human ovary. Scale bar, 50 μm. (E) Bar plot showing the relative number of STAR+ cells within the human ovary at ages 32, 34, and 42, compared to age 12. Two‐tailed Stud [file ACEL-25-e70288-s002.pdf]
